# Loss of Pum2 exacerbates colitis by disrupting macrophage–epithelial crosstalk and promoting epithelial necroptosis

**DOI:** 10.1038/s41420-026-03041-x

**Published:** 2026-03-20

**Authors:** Xuefei Wang, Xiaoxiao Han, Wenlin Qiu, Lijuan Jiang, Xiaoru Duan, Xiaojing Liu

**Affiliations:** 1 Union Hospital, Tongji Medical College, Huazhong University of Science and Technology, Wuhan, China; 2 Affiliated Hospital of Nantong University, Nantong, China

**Keywords:** Ulcerative colitis, Necroptosis

## Abstract

Ulcerative colitis (UC) is a chronic, relapsing inflammatory disorder characterized by persistent mucosal immune activation and compromised epithelial barrier function. In this study, we identify the RNA-binding protein PUMILIO2 (Pum2) as a previously unrecognized regulator of intestinal inflammation. Analysis of colonic tissues from UC patients revealed reduced Pum2 expression, which inversely correlated with disease activity. In dextran sulfate sodium (DSS)-induced colitis models, Pum2 deficiency exacerbated mucosal injury, accompanied by heightened macrophage inflammation. Mechanistically, Pum2 loss during colitis drives macrophage hyperactivation and TNFα-dependent epithelial necroptosis, which together intensify pathogenic macrophage–epithelial interactions and barrier breakdown. The dynamic downregulation of Pum2 in active inflammation underscores its potential as a therapeutic target for modulating macrophage–epithelial interactions and restoring intestinal barrier integrity in the context of colitis.

**Figure:**
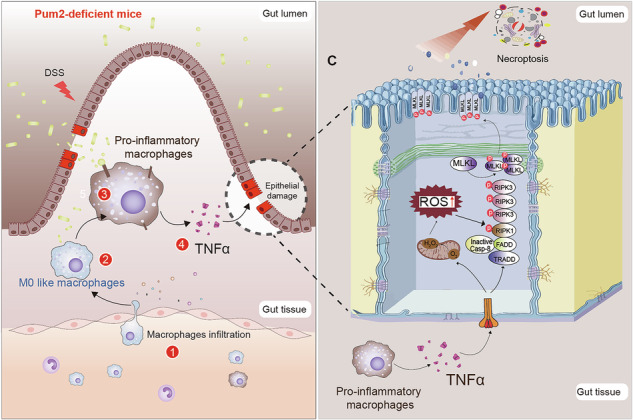
**Abstract Figure. Pum2 deficiency aggravates colitis via macrophage–epithelial crosstalk driving inflammation and necroptosis. Left**: Pum2 loss promotes macrophage-driven inflammation, with increased chemokine expression, macrophage infiltration, and a pro-inflammatory phenotype characterized by TNFα secretion. **Right**: Macrophage–epithelial crosstalk triggers epithelial necroptosis. Proinflammatory signals from Pum2-deficient macrophages sensitize epithelial cells to TNFα-induced death. Simultaneously, epithelial Pum2 loss elevates ROS, facilitating RIPK1, RIPK3, and MLKL phosphorylation. This synergistic cascade amplifies necroptosis and establishes a self-perpetuating loop of barrier disruption and inflammation.

**Abstract Figure. Pum2 deficiency aggravates colitis via macrophage–epithelial crosstalk driving inflammation and necroptosis. Left**: Pum2 loss promotes macrophage-driven inflammation, with increased chemokine expression, macrophage infiltration, and a pro-inflammatory phenotype characterized by TNFα secretion. **Right**: Macrophage–epithelial crosstalk triggers epithelial necroptosis. Proinflammatory signals from Pum2-deficient macrophages sensitize epithelial cells to TNFα-induced death. Simultaneously, epithelial Pum2 loss elevates ROS, facilitating RIPK1, RIPK3, and MLKL phosphorylation. This synergistic cascade amplifies necroptosis and establishes a self-perpetuating loop of barrier disruption and inflammation.

## Introduction

Ulcerative colitis (UC), a major subtype of inflammatory bowel disease (IBD), is characterized by chronic and recurrent inflammation of the colonic mucosa. Its increasing global incidence and relapsing clinical course pose a significant healthcare challenge, with patients facing heightened risks of life-threatening complications, including colorectal cancer (CRC) [1, 2]. Central to UC pathogenesis is the disruption of intestinal epithelial barrier integrity, coupled with aberrant immune activation, which perpetuates mucosal injury and chronic inflammation [3, 4]. The unpredictable onset and severity of disease flares further complicate management, highlighting the urgent need to elucidate the molecular mechanisms driving mucosal injury and inflammation.

RNA-binding proteins (RBPs) have recently emerged as key post-transcriptional regulators of gene expression, influencing mRNA splicing, stability, and translation. They play critical roles in both immune regulation and epithelial cell function [5, 6]. Among them, PUMILIO2 (Pum2), a member of the PUF family, has been implicated in diverse biological processes, including stem cell differentiation, metabolism, cell cycle control, and tumorigenesis [7–11]. Notably, Pum2 deficiency has been associated with mitochondrial dysfunction and excessive reactive oxygen species (ROS) generation [12, 13]—a hallmark of oxidative stress that contributes to epithelial damage and mucosal inflammation in colitis [14, 15]. Despite these observations, the direct role of Pum2 in colitis remains poorly defined.

Previous studies suggest that Pum2 loss may suppress colonic tumorigenesis under certain conditions [10]. Interestingly, chronic, relapsing inflammation in UC is a critical driver of colorectal cancer (CRC) development [16], and some molecules exhibit stage-dependent effects in both colitis and cancer, which may even appear opposing at different stages. For instance, deletion of STAT3 [17, 18], or TLR4 [19, 20] exacerbates DSS-induced colitis while suppressing colitis-associated tumorigenesis. A comprehensive focus on the roles of molecules in both inflammation and tumorigenesis offers a deeper understanding of the inflammation-cancer axis, further underscoring the need to investigate the role of Pum2 in acute intestinal inflammation.

In this study, we assessed Pum2 expression in human samples of IBD and CRC and investigated the mechanistic role of Pum2 in acute inflammation using Pum2-deficient (Pum2−/−) mice in the acute dextran sulfate sodium (DSS) colitis model, a well-established model that induces epithelial barrier disruption and early innate immune responses [21]. We systematically assessed clinical and histological indices of colitis severity, epithelial barrier integrity, and inflammatory cytokine production. In addition, a macrophage–epithelial co-culture system was employed to delineate the immunoregulatory functions of Pum2 across both immune and epithelial compartments. Our findings demonstrate that loss of Pum2 during colitis exacerbates macrophage-driven inflammation by enhancing TNFα release, while simultaneously rendering epithelial cells more susceptible to TNFα-induced necroptosis through impaired ROS control. These findings position Pum2 as a critical post-transcriptional checkpoint in the early inflammatory cascade, providing a foundation for future investigations into its role in chronic inflammation and cancer progression.

## Results

### Pum2 is downregulated in UC but reactivated in CRC: evidence from human cohorts and murine models

To clarify the pathological role of Pum2 in colitis and colitis-associated carcinogenesis, we systematically analyzed multiple transcriptomic datasets from human cohorts and murine models.

**Fig. 1:**
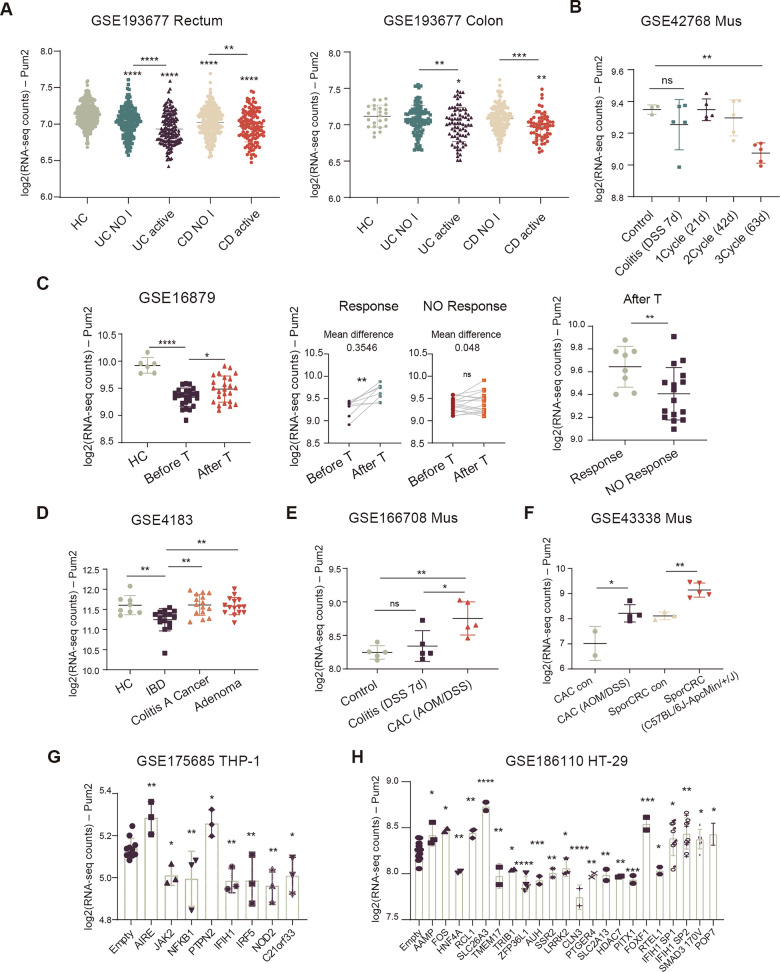
Altered expression patterns of Pum2 in IBD and CRC across human and murine models. **A** Pum2 expression in colonic/rectal biopsies from IBD patients and healthy controls (GSE193677). Colon-level effects were obtained by pooling the transverse, left, and right colon. One-way ANOVA with Tukey’s post-hoc test. ns: not significant; *P < 0.05; **P < 0.01; ****P < 0.0001. HC healthy controls, UC NO I non-inflamed UC patients. UC active active UC patients, CD NO I non-inflamed CD patients, CD active active CD patients. **B** Time-course analysis of Pum2 expression in DSS-treated mice colon (GSE42768). 1 cycle was defined as a 7-day DSS followed by 14-day water. Statistical significance was determined using One-way ANOVA, post-hoc Tukey’s test. ns: not significant; **P < 0.01. **C** Therapy-associated Pum2 regulation in UC (GSE16879). Left: Comparison of Pum2 expression before and after treatment (One-way ANOVA, Tukey). Middle: Pre-treatment and post-treatment Pum2 expression comparisons within responders and non-responders (paired t-test). Right: post-treatment responders vs non-responders (unpaired t-test). ns: not significant; *P < 0.05; **P < 0.01; ****P < 0.0001. HC: healthy controls; Before T: before treatment; After T: after treatment. **D** Comparative expression in IBD mucosa, colitis-associated tumors, and healthy controls (GSE4183). Statistical significance was assessed using one-way ANOVA with Tukey’s post-hoc test. **P < 0.01. **E**, **F** Validation of Pum2 induction in murine models of colitis-associated cancer (AOM/DSS) and spontaneous CRC (ApcMin/+) compared with colitis or control mucosa (GSE166708, GSE43338). One-way ANOVA with Tukey’s post-hoc test. ns: not significant; *P < 0.05; **P < 0.01. CAC colitis-associated cancer; SporCRC spontaneous CRC, con control. **G**, **H** Upstream regulatory analysis using ectopic expression of IBD-associated ORFs in THP-1 monocytes (GSE175685) and HT-29 epithelial cells (GSE186110). One-way ANOVA with Tukey’s post-hoc test. *P < 0.05; **P < 0.01; ***P < 0.001; ****P < 0.0001.

In the large IBD cohort GSE193677 (*n* = 2490 biopsies), Pum2 expression was reduced in both UC and CD, with the strongest suppression in the colon and rectum—the primary sites of UC pathology—and particularly in actively inflamed mucosa (Fig. 1A). In contrast, the cecum and ileum showed only nonsignificant downward trends relative to healthy controls, likely due to limited sample size (Supplementary Fig. 1A). Consistent with a cumulative effect of inflammation, murine colitis models (GSE42768) showed no change after a single DSS cycle but progressive downregulation with repeated cycles (Fig. 1B, Supplementary Fig. 1B).

Therapeutic cohorts further demonstrated dynamic regulation. In GSE16879, Pum2 was restored after 4–6 weeks of treatment in responders, but remained unchanged in non-responders (Fig. 1C). In the longer-term follow-up of the GSE186582 cohort, patients in sustained remission (REM) exhibited greater recovery (mean difference = 0.25) compared with relapsed cases (REC; mean difference = 0.12) after six months (Supplementary Fig. 1C). Together, these results indicate that Pum2 declines during active inflammation, increases with mucosal healing, and remains insufficiently restored in relapse-prone patients. However, overlapping expression distributions in GSE186582 suggest that Pum2 reflects short-term inflammatory activity more reliably than long-term prognosis.

In contrast, Pum2 was consistently reactivated during neoplastic progression. Previous studies have validated both the upregulation and functional activation of Pum2 in colorectal cancer [10]. In our study, Pum2 was reduced in inflamed IBD mucosa relative to healthy controls but significantly elevated in colitis-associated tumors compared with inflamed mucosa (Fig. 1D). Interestingly, its levels in colitis-associated tumors were not significantly different from those in healthy mucosa, which may reflect the biological characteristics of early-stage tumors and the inherent heterogeneity of tumorigenesis. This tumor-associated induction was further confirmed in murine AOM/DSS models (GSE166708), where robust Pum2 upregulation was observed in tumors relative to both colitis and control mucosa (Fig. 1E). Acute DSS exposure alone had only minimal changes compare to control, in line with our data (day 7 DSS in GSE166708, Fig. 1B) that short-term DSS injury alone does not alter Pum2. Similar tumor-associated upregulation was corroborated in GSE43338 (Fig. 1F). Collectively, these data delineate a stage-dependent trajectory in which Pum2 is suppressed during inflammation, partially restored upon mucosal remission, and reactivated during tumor development.

To investigate upstream mechanisms driving Pum2 dysregulation, we analyzed ORF-based datasets involving ectopic expression of IBD-associated genes in THP-1 and HT-29 cells (GSE175685, GSE186110). Several candidate genes modulating Pum2 expression were identified (Fig. 1G, H; Supplementary Fig. 2A, B), providing initial mechanistic insights into its transcriptional control in the inflamed mucosal environment.

### Pum2 deficiency exacerbates DSS-induced colitis and intestinal injury

As shown in Result 1, Pum2 expression is altered during intestinal inflammation, with a marked downregulation in active colitis patients. To investigate Pum2’s function in acute immune responses and epithelial barrier disruption, we used an acute DSS model. While Pum2 expression did not significantly change during the short induction period, this model is crucial for studying the early inflammatory processes, which are difficult to examine in chronic models. To explore this, we evaluated the effects of Pum2 deficiency using Pum2^−/−^ mice, which were validated by PCR genotyping and immunohistochemistry (Supplementary Fig. 3A, B).

**Fig. 2:**
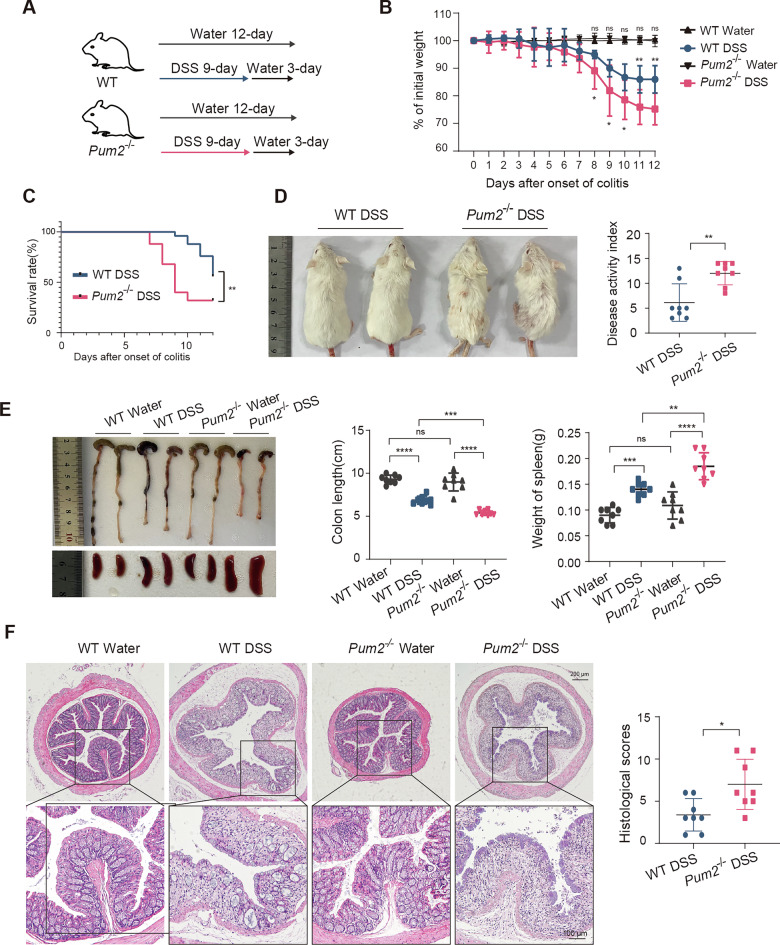
Pum2 deficiency aggravates DSS-induced colitis and enhances intestinal damage. **A** Experimental design illustrating the administration schedule of dextran sulfate sodium (DSS) for the induction of acute colitis in wild-type (WT) and Pum2-deficient (Pum2−/−) mice. **B** Longitudinal assessment of body weight changes in WT and Pum2−/− littermates during DSS exposure (*n* = 8). **P < 0.01. **C** Kaplan–Meier survival analysis comparing the survival probability of WT and Pum2−/− mice following either water or DSS treatment (*n* = 25). **P < 0.01. **D** Representative images and quantitative scoring of Disease Activity Index (DAI), including weight loss, stool consistency, and rectal bleeding, in WT and Pum2−/− mice under control or DSS-treated conditions (*n* = 8). **P < 0.01. **E** Macroscopic appearance of colonic and splenic tissues from WT and Pum2−/− mice after water or DSS exposure. Bar graphs summarize colon length and spleen weight (*n* = 8). ns: not significant; **P < 0.01; ***P < 0.001; ****P < 0.0001. **F** Representative hematoxylin and eosin (H&E) staining of colonic sections illustrating histological features of inflammation, epithelial damage, and immune cell infiltration in WT and Pum2−/− mice. Quantification of histopathological scores post-DSS treatment (*n* = 8). Scale bar = 100 μm. *P < 0.05.

Both WT and Pum2^−/−^ littermates received 2.5% DSS for 9 days followed by 3 days of recovery (Fig. 2A). While DSS exposure induced weight loss in both groups, Pum2−/− mice demonstrated significantly greater body mass reduction starting at day 8 relative to WT controls (Fig. 2B). Furthermore, survival analysis revealed a marked decrease in the survival rate of Pum2−/− mice compared to WT mice (Fig. 2C). Phenotypically, Pum2−/− mice exhibited a more pronounced disease activity index including reduced activity, weight loss, and rectal bleeding (Fig. 2D).

Consistent with these findings, Pum2−/− mice displayed greater colon shortening and splenomegaly (Fig. 2E), indicative of enhanced systemic inflammation. Histological examination revealed extensive epithelial disruption, crypt architectural distortion, and increased infiltration of immune cells in Pum2−/− colons (Fig. 2F). Together, these results demonstrate that Pum2 deficiency aggravates DSS-induced intestinal injury and inflammation.

### Transcriptomic profiling identifies chemokine-driven immune priming in Pum2-deficient colonic tissue

As Pum2 is an RNA-binding protein central to post-transcriptional regulation, we hypothesized that its loss might induce latent transcriptional alterations even in the absence of overt pathology. Although WT and Pum2−/− mice under basal conditions showed no differences in body weight, colon length, or spleen weight, we performed baseline RNA-seq on untreated colonic tissues to capture hidden molecular changes without the confounding effects of DSS-induced reprogramming [22]. Similar strategies in other RBP-deficient models (RBM47, TTP) have revealed immune-regulatory programs predisposing animals to colitis despite minimal baseline phenotypes [23, 24].

**Fig. 3:**
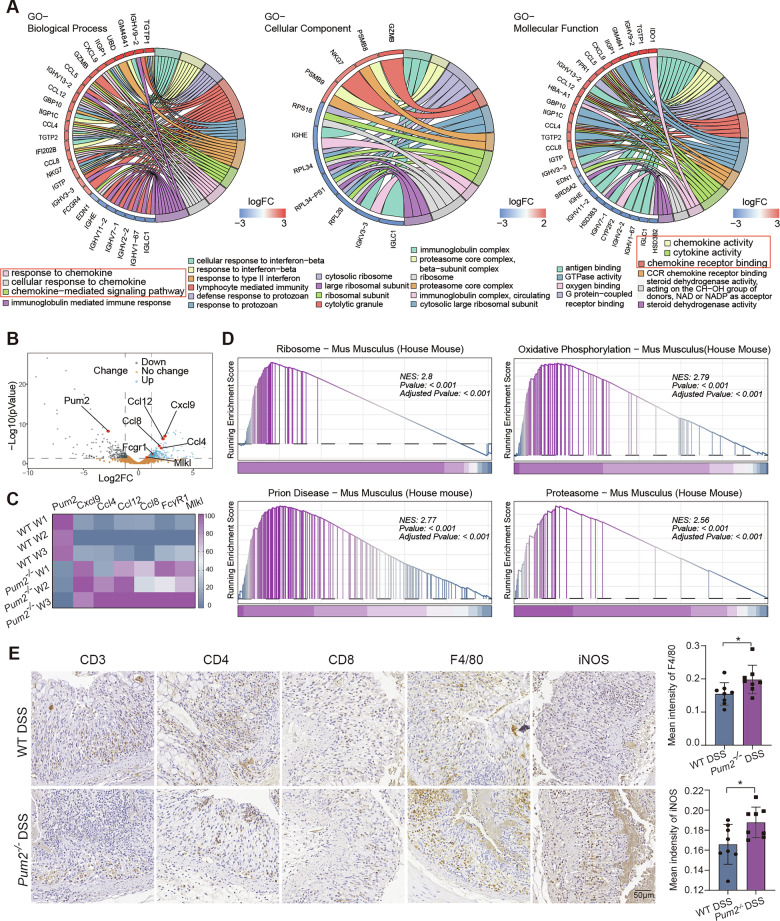
Transcriptomic profiling and immune-cell infiltration in colonic tissues of Pum2−/− mice. **A** Gene Ontology (GO) enrichment analysis of differentially expressed genes (DEGs) identified in colonic tissues from Pum2−/− versus wild-type (WT) mice, with categorization into biological processes (BP), molecular functions (MF), and cellular components (CC). **B** Volcano plot showing the distribution of DEGs, with log₂ fold changes and statistical significance differentiating upregulated and downregulated genes between Pum2−/− and WT mice. **C** Heatmap illustrating the expression patterns of seven representative DEGs that are markedly dysregulated in Pum2−/− (water) mice compared with WT (water) controls. WT W: WT mice treated with water; Pum2−/− W: Pum2−/− mice treated with water. **D** Gene Set Enrichment Analysis (GSEA) identifies pathways significantly enriched in Pum2−/− colonic tissues, highlighting enhanced inflammatory and immune-related gene signatures. **E** IHC and quantification of CD3, CD4, CD8 (T lymphocyte markers), F4/80 (macrophage marker), and iNOS (pro-inflammatory marker) in colon sections from Pum2−/− and WT mice treated with DSS (*n* = 8). Scale bar = 50 μm. *P < 0.05.

At baseline, Gene Ontology analysis showed selective enrichment of pathways related to chemokine activity and leukocyte trafficking (Fig. 3A). Differential expression highlighted upregulation of Ccl4, Ccl8, and Ccl12—potent drivers of monocyte/macrophage recruitment—as well as Cxcl9, a marker of M1-like macrophage polarization (Fig. 3A–C). Complementary GSEA indicated enrichment of ribosome biogenesis, proteasomal processing, prion-associated pathways, and oxidative phosphorylation (Fig. 3D), suggesting subtle metabolic rewiring that may facilitate rapid inflammatory activation in the absence of Pum2.

Despite these transcriptional shifts, baseline immune infiltration showed no differences between WT and Pum2−/− mice. IHC revealed minimal CD3, CD4, CD8, and F4/80 staining, and RNA-seq/qPCR confirmed no changes in barrier- or infiltration-related signatures (Fig. 3B; Supplementary Fig. 3C, 4B). Thus, Pum2 deficiency establishes a chemokine-biased transcriptional program that preserves homeostasis at rest but sensitizes tissue to inflammatory challenge.

Upon DSS exposure, both genotypes exhibited robust immune cell infiltration, but macrophage responses were disproportionately amplified in Pum2−/− mice, as shown by increased F4/80+ cells and elevated iNOS expression (Fig. 3E; Supplementary Fig. 4A, B). These findings indicate that Pum2 loss selectively enhances macrophage recruitment and polarization toward a pro-inflammatory phenotype, a hallmark of UC pathology [25].

Single-cell RNA-seq (GSE148794) provided further context. At baseline, Pum2 was enriched in colonocytes, endothelial cells, and macrophages. After DSS (6 days) and recovery (6 days), all three compartments showed no pronounced reduction in Pum2, consistent with the limited impact of short-term DSS in bulk datasets (Fig. 1B; Supplementary Fig. 4C). Collectively, these results emphasize the importance of investigating macrophages and epithelial cells in shaping the inflammatory dynamics of Pum2−/− mice.

### Pum2 deficiency augments pro-inflammatory macrophage activation

Aberrant immune activation, particularly by macrophages, is a hallmark of UC pathogenesis [26]. Monocyte-derived macrophages infiltrate the inflamed mucosa and secrete cytokines such as TNFα, IL1β, and IL6, which exacerbate epithelial damage and sustain chronic inflammation [27–29]. Given the pronounced macrophage infiltration observed in Pum2−/− colons during colitis, we next examined whether Pum2 regulates macrophage inflammatory responses in vitro.

**Fig. 4:**
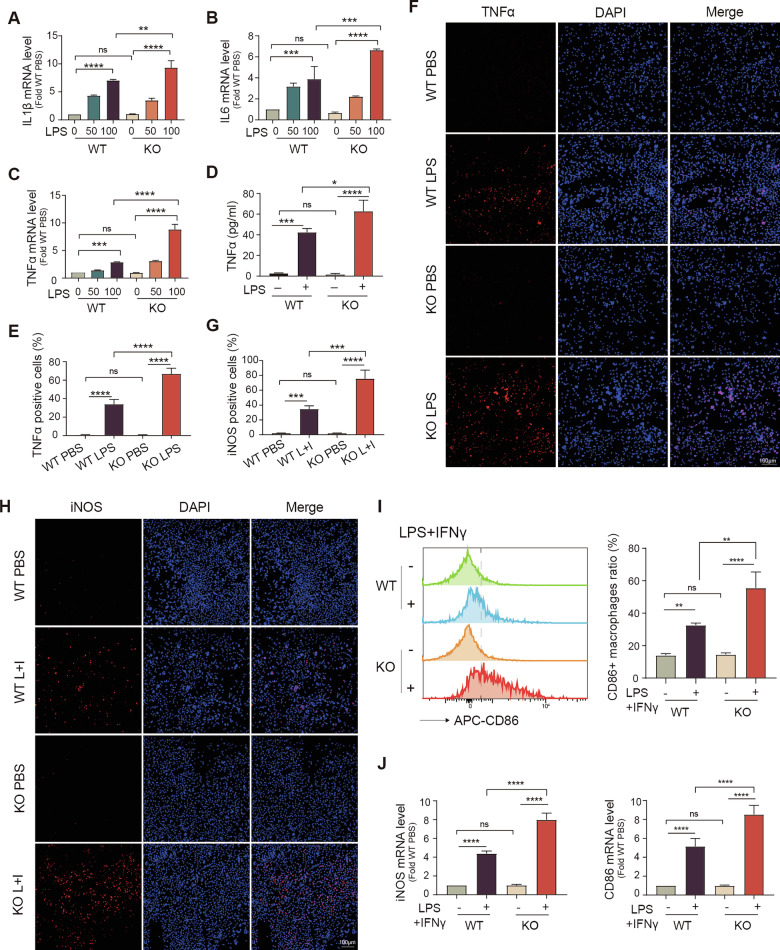
Pum2 deficiency promotes a hyperinflammatory macrophage phenotype with enhanced cytokine production. **A**–**C** Quantitative analysis of IL1β, IL6, and TNFα mRNA expression in BMDMs isolated from WT and Pum2−/− mice under basal and LPS-stimulated (50 or 100 ng/mL, 6 h) conditions. One-way ANOVA with Tukey’s post-hoc test. ns: not significant; *P < 0.05; **P < 0.01; ***P < 0.001; ****P < 0.0001. WT: WT BMDMs; KO: Pum2−/− BMDMs. **D** ELISA measurement of TNFα protein levels in culture supernatants of BMDMs treated with LPS (100 ng/mL) for 24 h. One-way ANOVA with Tukey’s post-hoc test. ns: not significant; *P < 0.05; ***P < 0.001; ****P < 0.0001. **E**, **F** Immunofluorescent staining and quantification of TNFα-positive BMDMs (red) following LPS stimulation (100 ng/mL, 6 h), with brefeldin A (BFA, 1 μg/mL) added for the final 3 h to block cytokine secretion. Scale bar = 100 μm. One-way ANOVA with Tukey’s post-hoc test. ns: not significant; ****P < 0.0001. **G**, **H** Immunofluorescence detection and quantification of iNOS-expressing macrophages (red) after treatment with LPS (100 ng/mL) and IFNγ (25 ng/mL) for 24 h. Scale bar = 100 μm. One-way ANOVA with Tukey’s post-hoc test. ns: not significant; ***P < 0.001; ****P < 0.0001. **I** Flow cytometric analysis of CD86 surface expression (an M1 macrophage marker) in BMDMs from WT and Pum2−/− mice following M1 polarization with LPS and IFNγ for 24 h. One-way ANOVA with Tukey’s post-hoc test. ns: not significant; **P < 0.01; ***P < 0.001. **J** mRNA expression levels of M1-associated markers Cd86 and Nos2 (encoding iNOS) in WT and Pum2-deficient BMDMs after LPS/IFNγ stimulation for 24 h. One-way ANOVA with Tukey’s post-hoc test. ns not significant; ****P < 0.0001.

Bone marrow–derived macrophages (BMDMs) from WT and Pum2−/− mice were stimulated with lipopolysaccharide (LPS). Compared with WT cells, Pum2-deficient macrophages exhibited markedly elevated transcription of TNFα, IL6, and IL1β (Fig. 4A–C). Notably, TNFα, a pivotal cytokine in UC progression and therapeutic target [30], was profoundly upregulated in Pum2−/− macrophages, underscoring its pathological relevance. Consistent with mRNA expression levels, ELISA assays demonstrated increased TNFα secretion in the culture supernatants of Pum2−/− BMDMs (Fig. 4D), and immunofluorescence showed enhanced intracellular TNFα protein after LPS challenge (Fig. 4E, F).

Phenotypic profiling of the macrophages revealed a skewing toward a classically activated, M1-like phenotype in the absence of Pum2. Flow cytometry, RT-qPCR, and immunostaining collectively showed an increased proportion of CD86+ and iNOS+ macrophages in the Pum2 − /− group (Fig. 4G–J), indicating enhanced polarization toward a pro-inflammatory state. Conversely, Pum2 overexpression in macrophage cell lines suppressed LPS-induced pro-inflammatory cytokine production, particularly TNFα, and reduced expression of M1-associated markers CD86 and iNOS (Supplementary Fig. 5A–G).

Together, these findings identify Pum2 as a negative regulator of macrophage activation. Loss of Pum2 amplifies pro-inflammatory cytokine production and promotes M1 polarization, indicating that Pum2 is critical for maintaining macrophage homeostasis during intestinal inflammation.

### Elevated TNFα expression and TNFα-mediated epithelial injury in Pum2-deficient mice with DSS-induced colitis

**Fig. 5:**
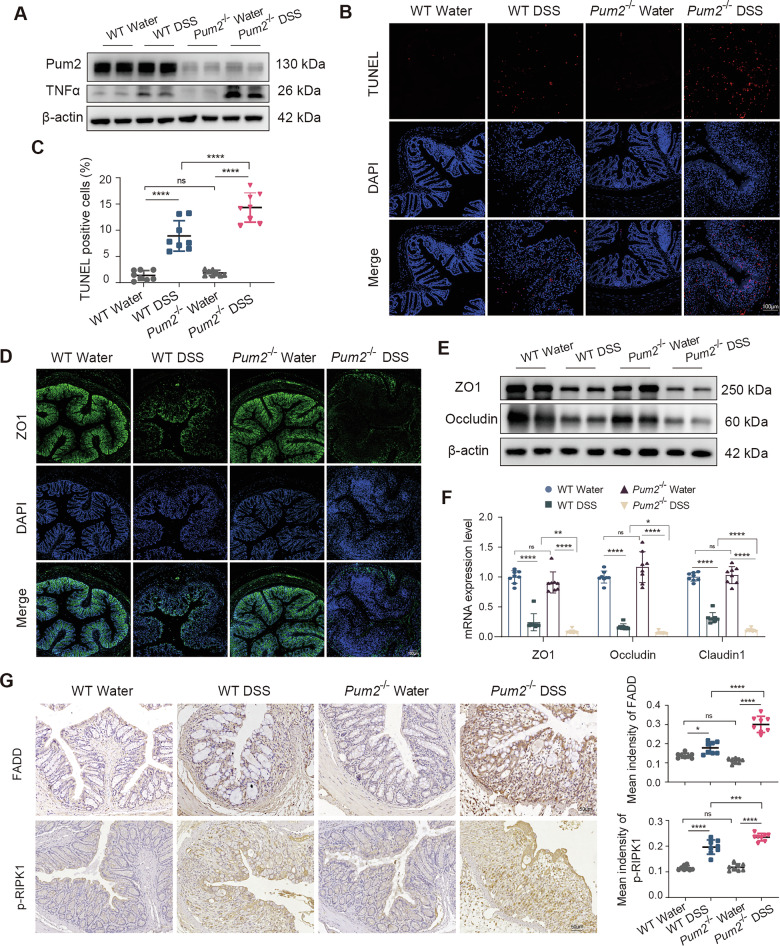
Pum2 deficiency amplifies TNFα-mediated inflammatory signaling and epithelial cell death in DSS-induced colitis. **A** WB analysis of TNFα protein levels in colonic tissues from WT and Pum2−/− mice following either water or DSS administration. **B** Representative TUNEL staining images (red) illustrating epithelial apoptosis/necrosis in colonic sections from WT and Pum2−/− mice treated with water or DSS. Scale bar = 100 μm. **C** Quantification of TUNEL-positive cells per high-power field in colonic tissues of WT and Pum2−/− mice treated with water or DSS (*n* = 8). One-way ANOVA with Tukey’s post-hoc test. ns: not significant; ****P < 0.0001. **D** Immunofluorescent staining of tight junction protein ZO1 in colonic tissues of WT and Pum2−/− mice treated with water or DSS. Scale bar = 100 μm. **E** Western blotting of ZO1 and Occludin in colonic lysates from WT and Pum2−/− mice treated with water or DSS, assessing epithelial barrier integrity. **F** qPCR analysis of ZO1, Occludin and Claudin1 in colonic tissues from WT and Pum2−/− mice treated with water or DSS. One-way ANOVA with Tukey’s post-hoc test (*n* = 8). ns: not significant; *P < 0.05; **P < 0.01; ****P < 0.0001. **G** IHC and quantification of FADD and phosphorylated RIPK1 (p-RIPK1) in colonic sections (*n* = 8). Scale bar = 50 μm. One-way ANOVA with Tukey’s post-hoc test. ns: not significant; *P < 0.05; ***P < 0.001; ****P < 0.0001.

Building on the enhanced TNFα production observed in Pum2-deficient macrophages, we examined whether this hyperinflammatory state manifests in vivo during DSS-induced colitis. Under baseline water-fed conditions, both genotypes showed minimal cytokine expression, which was below the detection threshold of qPCR and therefore not included in the data. After DSS exposure, however, Pum2 − /− mice exhibited markedly higher colonic mRNA levels of TNFα, IL6, and IL1β compared with WT controls (Supplementary Fig. 6A). Consistently, Western blotting demonstrated increased TNFα protein in Pum2−/− colonic lysates (Fig. 5A; Supplementary Fig. 6B), and immunofluorescence confirmed enhanced TNFα localization in inflamed mucosa (Supplementary Fig. 6C).

Given TNFα’s central role in driving epithelial injury via programmed cell death [31], we next assessed epithelial integrity. TUNEL staining revealed significantly increased epithelial apoptosis/necrosis in DSS-treated Pum2−/− colons compared with WT mice (Fig. 5B, C).

To determine whether this injury compromised barrier function, we evaluated tight junction proteins. Under baseline conditions, ZO1 and Occludin were comparable between groups. Following DSS, however, both proteins were markedly reduced in Pum2−/− mice, as shown by immunofluorescence and Western blotting (Fig. 5D, E; Supplementary Fig. 6D, E). qPCR analysis also demonstrated decreased ZO1, Occludin, and Claudin1 transcripts (Fig. 5F). Despite this loss, Ki67 staining revealed no difference in epithelial proliferation (Supplementary Fig. 6F), indicating that impaired barrier function arose from enhanced epithelial death rather than defective regeneration.

Mechanistically, we observed increased expression of TNFα-induced cell death-associated mediators, including Fas-associated death domain (FADD) and phosphorylated receptor-interacting protein kinase 1 (p-RIPK1) [32] (Fig. 5G). These findings suggest that intensified epithelial injury in Pum2-deficient mice is driven by overactivation of TNFα-mediated apoptotic and necroptotic pathways.

### Pum2 deficiency sensitizes intestinal epithelial cells to TNFα-induced necroptosis via ROS accumulation

RNA-binding proteins (RBPs) regulate diverse cellular processes in a context-dependent manner. Pum2 has previously been implicated in controlling p21 (CDKN1A) expression in intestinal epithelial cells (IECs) [10]. Transcriptomic profiling of Pum2−/− colons revealed enrichment of oxidative phosphorylation (OXPHOS) pathways (Fig. 3D), a metabolic state often linked to mitochondrial reactive oxygen species (ROS) production [33]. Excessive ROS impairs barrier integrity and promotes epithelial death, contributing to mucosal injury in colitis [34].

**Fig. 6:**
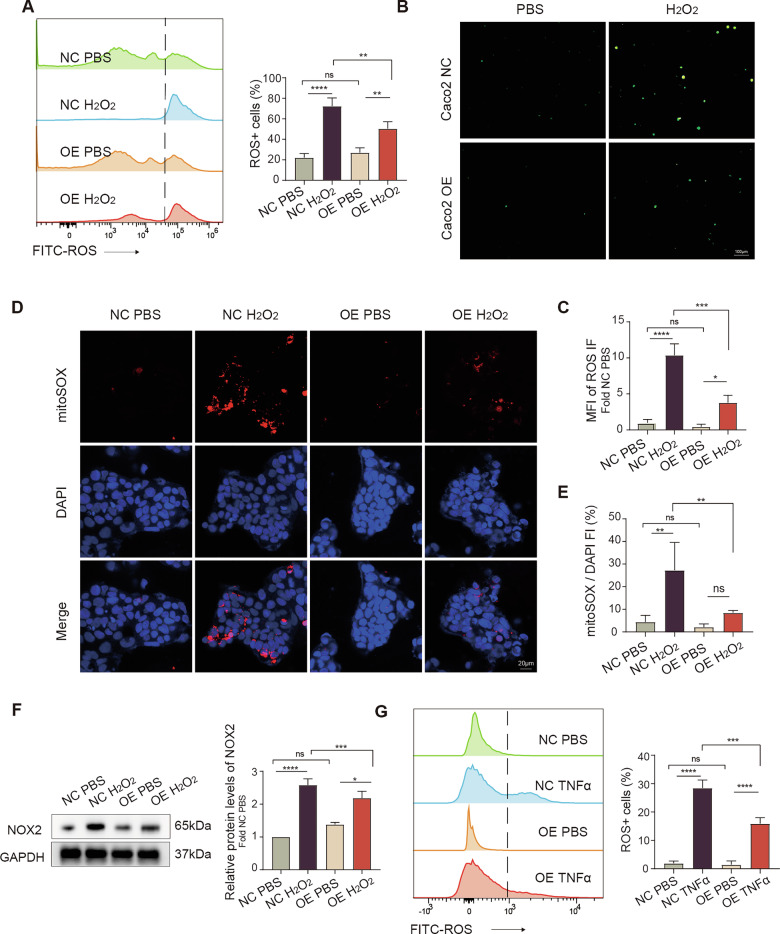
Pum2 overexpression mitigates oxidative stress and reduces intracellular ROS in Caco2 cells. **A** Flow cytometric quantification of intracellular ROS (DCF fluorescence) in OE-NC and OE-Pum2 Caco2 cells pretreated with PBS or H2O2 (500 μM, 4 h). One-way ANOVA with Tukey’s post-hoc test. ns: not significant; **P < 0.01; ****P < 0.0001. NC PBS: OE-NC Caco2 + PBS; NC H2O2: OE-NC Caco2 + H2O2; OE PBS: OE-Pum2 Caco2 + PBS; OE H2O2: OE-Pum2 Caco2 + H2O2. **B**, **C** Fluorescence microscopy and mean fluorescence intensity (MFI) quantification of intracellular ROS in H2O2-treated Caco2 cells. Scale bar = 100 μm. One-way ANOVA with Tukey’s post-hoc test. ns: not significant; *P < 0.05; ***P < 0.001; ****P < 0.0001. Caco2 NC: OE-NC Caco2 cells; OE: OE-Pum2 Caco2 cells. (**D**–**E**) MitoSOX™ Red staining of mitochondrial ROS with representative images and quantification. Scale bar = 20 μm. One-way ANOVA with Tukey’s post-hoc test. ns: not significant; **P < 0.01. **F** Western blot analysis of NADPH oxidase 2 (NOX2) protein expression in Caco2 cells following 24-hour H2O2 stimulation. One-way ANOVA with Tukey’s post-hoc test. ns: not significant; *P < 0.05; ***P < 0.001; ****P < 0.0001. **G** Flow cytometric assessment of TNFα-induced ROS (100 ng/mL, 2 h). One-way ANOVA with Tukey’s post-hoc test. ns: not significant; ***P < 0.001; ****P < 0.0001.

To test whether Pum2 modulates ROS accumulation, we quantified intracellular ROS in IECs under oxidative stress. Following H2O2 stimulation, Pum2 overexpression (OE) in Caco2 cells markedly reduced ROS levels, as shown by flow cytometry and fluorescence imaging (Fig. 6A–C). MitoSOX assays confirmed decreased mitochondrial ROS (Fig. 6D–E). Since NADPH oxidases, particularly NOX2, are key drivers of cytosolic ROS production, we examined NOX2 expression and found that Pum2-OE attenuated H2O2-induced NOX2 upregulation (Fig. 6F). Pum2-OE also suppressed TNFα-induced ROS generation (Fig. 6G), indicating a key role for Pum2 in limiting oxidative stress in IECs during colitis.

**Fig. 7:**
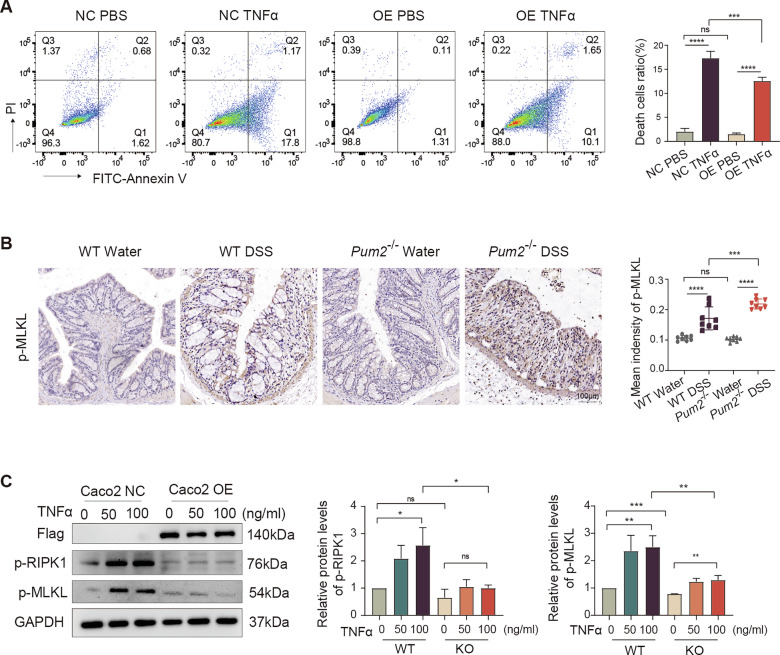
Pum2 modulates TNFα-induced phosphorylation of RIPK1 and MLKL in epithelial cells. **A** Annexin V/PI flow cytometry of apoptosis/necrosis in Caco2 cells transfected with OE-NC (NC) or Pum2 overexpression (OE) and stimulated with TNFα (100 ng/mL, 2 h); quantitative assessment shown. One-way ANOVA with Tukey’s post-hoc test. ns: not significant; ***P < 0.001; ****P < 0.0001. NC PBS: OE-NC Caco2 + PBS; NC TNFα: OE-NC Caco2 + TNFα; OE PBS: OE-Pum2 Caco2 + PBS; OE TNFα: OE-Pum2 Caco2 + TNFα. **B** IHC and quantification of phosphorylated MLKL (p-MLKL) in colonic sections from WT and Pum2−/− mice treated with either water or DSS (*n* = 8). Scale bar = 100 μm. One-way ANOVA with Tukey’s post-hoc test. ns: not significant; ***P < 0.001; ****P < 0.0001. **C** Western blots of phosphorylated RIPK1 and MLKL in NC and OE Caco2 cells treated with increasing TNFα concentrations (2 h). One-way ANOVA with Tukey’s post-hoc test. ns: not significant; *P < 0.05; **P < 0.01.

We next assessed susceptibility to TNFα-induced cell death. Annexin V/PI staining revealed that Pum2-OE significantly reduced apoptosis and necrosis in IECs (Fig. 7A). Mechanistically, ROS overproduction is known to promote TNFα-mediated necroptosis through phosphorylation of receptor-interacting protein kinase 1 (RIPK1) and mixed lineage kinase domain-like protein (MLKL) [35–37], with p-MLKL oligomerization and membrane translocation driving necroptotic lysis [32]. Consistent with this, MLKL expression was modestly upregulated in Pum2−/− transcriptomes (Fig. 3B), and IHC confirmed increased p-MLKL in DSS-treated Pum2−/− colons (Fig. 7B). Western blotting further showed that Pum2-OE suppressed TNFα-induced phosphorylation of RIPK1 and MLKL (Fig. 7C), directly linking Pum2 to inhibition of necroptotic signaling.

Collectively, under inflammatory stimulation, Pum2 deficiency promotes ROS accumulation and sensitizes IECs to TNFα-induced necroptosis, thereby exacerbating barrier disruption and inflammatory injury during colitis.

### Macrophage–epithelial co-culture reveals TNFα-dependent cross-talk driving necroptosis and barrier dysfunction

**Fig. 8:**
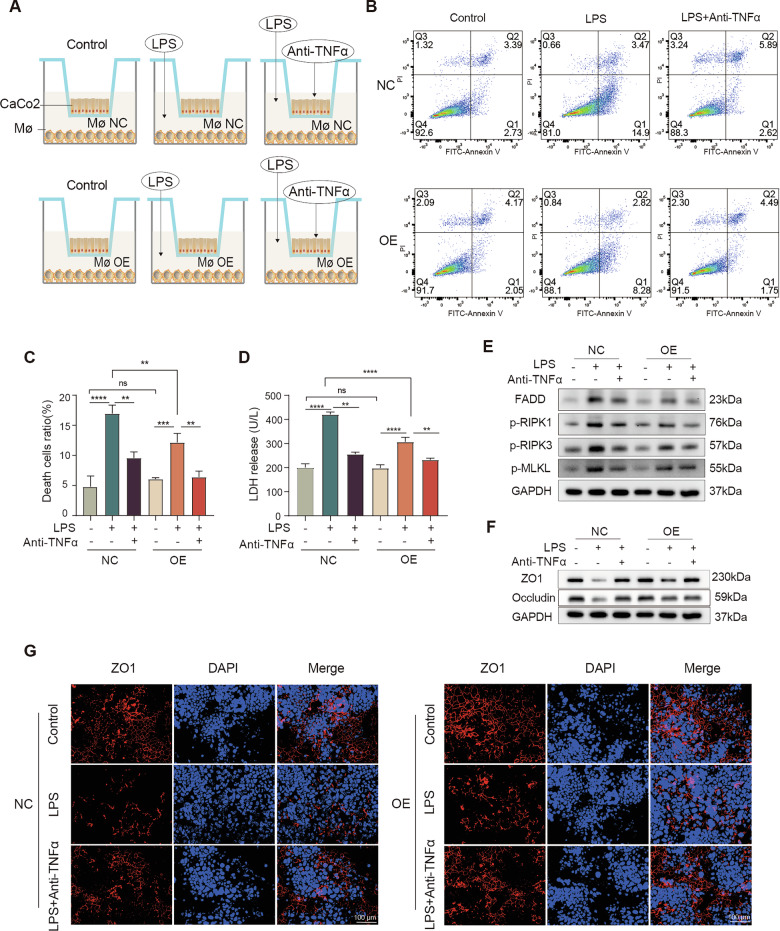
Co-culture shows Pum2 overexpression restrains TNFα-driven epithelial necroptosis and barrier loss. **A** Transwell co-culture schematic. THP-1–derived macrophages (Mφ; OE-NC or OE-Pum2) were LPS-stimulated (100 ng/mL, 24 h) with/without neutralizing anti-human TNFα (5 μg/mL). Caco2 epithelial monolayers were seeded in the upper chamber and co-cultured with either OE-NC THP-1 macrophages (Mφ-NC) or OE-Pum2 THP-1 macrophages (Mφ-OE). **B**, **C** Flow cytometric assessment of apoptosis/necrosis in Caco2 cells post co-culture. **B** Representative Annexin V/PI staining plots. **C** Quantification of total Caco2 cell death under indicated conditions. One-way ANOVA with Tukey’s post-hoc test. ns: not significant; **P < 0.01; ***P < 0.001; ****P < 0.0001. NC: Caco2 cells co-cultured with OE-NC THP-1 cells; OE: Caco2 cells co-cultured with OE-Pum2 THP-1 cells. **D** Measurement of lactate dehydrogenase (LDH) release as an indicator of Caco2 cytotoxicity. One-way ANOVA with Tukey’s post-hoc test. ns: not significant; **P < 0.01; ****P < 0.0001. **E** Western blots of necroptosis signaling (p-RIPK1, p-RIPK3, p-MLKL, FADD) in Caco2 cells after co-culture. **F** Western blot evaluation of epithelial tight junction proteins ZO1 and Occludin to assess the structural integrity of the epithelial barrier. **G** Immunofluorescence showing localization of ZO1 (red) in Caco2 cells under co-culture conditions. Nuclei were counterstained with DAPI (blue). Scale bar = 100 μm.

To model the inflammatory mucosal microenvironment, we established a transwell co-culture system enabling soluble factor–mediated communication between macrophages and epithelial cells. Caco2 epithelial monolayers were seeded in the upper chamber to form a polarized barrier, while THP-1–derived macrophages (either overexpressing Pum2 [OE] or transfected with control vector [NC]) were stimulated with LPS and placed in the lower chamber. A TNFα-neutralizing antibody was applied to determine the specific contribution of TNFα signaling (Fig. 8A).

Flow cytometry showed a significant increase in Annexin V/PI-positive epithelial cells when co-cultured with LPS-stimulated NC macrophages, indicating enhanced epithelial death. This cytotoxicity was markedly reduced by Pum2 overexpression in macrophages or TNFα neutralization (Fig. 8B, C). Consistently, LDH release from Caco2 cells—a marker of membrane integrity loss—was significantly reduced under both conditions (Fig. 8D), supporting a protective effect of Pum2 and TNFα blockade.

Mechanistically, epithelial cells exposed to NC macrophages displayed robust activation of necroptotic signaling, with increased phosphorylation of RIPK1, RIPK3, MLKL, and FADD. These effects were suppressed by Pum2 overexpression or TNFα inhibition (Fig. 8E). These findings reveal a critical axis of macrophage-to-epithelial communication in promoting inflammatory cell necroptosis. Finally, barrier integrity assays showed substantial downregulation of ZO1 and Occludin in epithelial cells co-cultured with NC macrophages, whereas Pum2-OE macrophages or TNFα blockade preserved tight junction expression (Fig. 8F, G).

Together, these results establish a pathogenic inflammatory loop in which macrophage-derived TNFα drives epithelial necroptosis and barrier disruption, and demonstrate that Pum2 restrains this macrophage–epithelial cross-talk during colitis.

## Discussion

The intestinal mucosa relies on a dynamic interplay between immune cells, epithelial cells, and the microbiota to maintain homeostasis and resist injury [26]. In this study, we identified a pivotal role for the RNA-binding protein PUMILIO2 (Pum2) in regulating intestinal inflammation. Our findings demonstrate that Pum2 is a key mediator of macrophage–epithelial cross-talk.

The acute DSS model has provided critical insights into Pum2’s role in modulating immune responses and epithelial injury, laying the foundation for future investigations in chronic inflammation. This approach, which links chronic clinical or genetic data to acute experimental models, is commonly used to uncover underlying molecular mechanisms [38–40]. In the acute phase of inflammation, Pum2 loss amplifies macrophage-driven inflammatory responses and exacerbates epithelial damage. Specifically, the absence of Pum2 accelerates chemokine expression and macrophage influx, aligning with the concept that colonic macrophages are replenished by circulating monocytes during colitis [28, 41]. These macrophages adopt a pro-inflammatory phenotype, secreting large amounts of cytokines such as TNFα and IL1β, and display a transcriptional profile resembling UC monocytes [42]. The resultant increase in cytokine release leads to sustained epithelial injury and disruption of the intestinal barrier.

We provided direct evidence for macrophage–epithelial cross-talk using our co-culture model. LPS-stimulated macrophages induced significant epithelial cell death, which was markedly reduced by Pum2 overexpression or TNFα neutralization. Downstream, intestinal epithelial cells (IECs) exhibited activation of RIPK1- and MLKL-dependent necroptosis, a lytic cell death pathway increasingly recognized as a key driver of epithelial erosion in colitis [43, 44]. In Pum2-deficient mice, predominant necroptotic IEC loss impaired regeneration and prolonged inflammation, thereby worsening DSS colitis [31].

Our findings also reveal a Pum2–ROS–necroptosis axis. Transcriptomic profiling indicated enrichment of oxidative phosphorylation in Pum2-deficient colons, a hallmark of mitochondrial dysfunction. Functional assays showed that Pum2 restrains ROS accumulation under inflammatory stimuli such as TNFα or H2O2. In its absence, excessive ROS amplified necroptotic signaling via RIPK1 and MLKL phosphorylation, thereby linking mitochondrial stress to epithelial barrier failure [45, 46].

Interestingly, despite no overt baseline phenotype, RNA-seq revealed a chemokine-biased transcriptional program in Pum2-deficient mice. This “latent priming” sensitized tissues to inflammatory challenge, paralleling IBD susceptibility genes such as XBP1, ATG16L1, and the RNA-binding protein HuR, which appear normal under homeostasis but increase disease risk under stress [38, 47–49]. Thus, Pum2 functions as a molecular checkpoint, preserving mucosal resilience at rest while constraining inflammatory escalation during challenge.

Several limitations should be noted. Most of the data in this study are based on transcriptomic analyses and acute DSS models, which do not fully capture the chronic nature of human IBD. Future studies using multi-cycle DSS or chronic models will be essential for better understanding Pum2’s role in chronic IBD. The contribution of the microbiota—an established driver of intestinal inflammation—was not addressed. Moreover, while our results suggest opposing roles for Pum2 in colitis and cancer, validation in clinical samples is required. Finally, we employed global Pum2 knockout mice, which may not faithfully reflect the partial and context-dependent downregulation observed in patients. More refined approaches, such as conditional or graded knockout models, will be essential to dissect cell–type–specific functions of Pum2 and to achieve more accurate disease modeling.

## Conclusions

This study identifies Pum2 as a critical regulator of intestinal immune–epithelial homeostasis during colitis. Pum2 loss aggravates DSS-induced colitis by amplifying macrophage-driven inflammation, triggering TNFα-dependent necroptosis in epithelial cells, and disrupting barrier integrity. Mechanistically, Pum2 restrains pro-inflammatory cytokine release and limits ROS-driven cell death pathways, thereby mitigating pathogenic macrophage–epithelial cross-talk. Clinically, Pum2 expression correlates with disease activity and recovery in colitis, suggesting its potential as a biomarker and therapeutic target for mucosal restoration.

## Methods

### Acquisition and analysis of transcriptomic data

To assess Pum2 expression across gastrointestinal inflammatory and neoplastic conditions, publicly available transcriptomic datasets were retrieved from the NCBI Gene Expression Omnibus (GEO). Datasets included patients with inflammatory bowel disease (IBD), ulcerative colitis (UC), Crohn’s disease (CD), and colorectal cancer (CRC). Murine datasets were included to complement human findings and to capture Pum2 dynamics under controlled experimental paradigms of colitis and tumorigenesis. The following GEO datasets were analyzed: GSE193677, GSE186582, GSE16879, GSE4183, GSE42768, GSE166708, GSE43338, and GSE148794. Data processing, normalization, and differential expression analyses were performed in R v4.2.0 and relevant Bioconductor packages. Group comparisons were defined according to metadata annotations. In addition, ORF-based datasets (GSE175685, GSE186110) were examined to identify upstream regulators of Pum2 expression in THP-1 and HT-29 cells.

### Mouse models

Pum2-knockout (Pum2−/−) mice were kindly provided by Professor Eugene Yujun Xu (Nanjing Medical University) [7] and maintained on an FVB background through serial backcrossing with wild-type FVB mice. Genotypes were confirmed by PCR, and knockout efficiency was validated by qPCR and IHC. All mice were housed under specific pathogen-free (SPF) conditions at the Animal Resource Centre of Tongji Medical College, Huazhong University of Science and Technology. All animal experiments were approved by the Institutional Animal Care and Use Committee (IACUC No. 4349) of Huazhong University of Science and Technology.

### Cell culture and treatment protocols

Primary bone marrow-derived macrophages (BMDMs) were harvested from the femoral and tibial bones of either wild-type or Pum2 knockout (Pum2−/−) mice and cultured in α-MEM supplemented with 30 ng/mL M-CSF, 10% FBS, and 1% penicillin–streptomycin. RAW264.7 murine macrophages were maintained in DMEM with 10% FBS and 1% penicillin–streptomycin. Macrophage polarization toward the M1 phenotype was induced by treatment with LPS (100 ng/mL) and IFNγ (25 ng/mL) for 24 h. Caco2 cells were cultured in RPMI 1640 supplemented with 10% FBS and 1% penicillin–streptomycin. For inflammatory activation assays, cells were treated with LPS (100 ng/mL) for 30 min or 24 h, depending on the experimental design.

### Statistical analysis

All data are presented as the mean ± standard deviation (SD). Comparisons between two groups were performed using paired or unpaired Student’s t-tests, as appropriate. Multiple group comparisons were conducted by one-way ANOVA, followed by Dunnett’s post hoc test (for comparisons against a single control) or Tukey’s multiple comparisons test (for all pairwise comparisons). For non-parametric data, the Mann–Whitney U test or Kruskal–Wallis test with Dunn’s post hoc correction was applied. Kaplan–Meier survival curves were analyzed using the log-rank test. All statistical analyses were performed using GraphPad Prism v8.0 and R v4.2.0. A *P* value < 0.05 was considered statistically significant.

Detailed experimental protocols, including DSS treatment schedules, antibody information, flow cytometry procedures, and western blot analysis, are provided in the Supplementary Methods.

## Supplementary information

Supplementary Methods

Supplementary Figures 1-6.

Supplementary Table1.

Supplementary Table2.

WB raw data

## Data Availability

The transcriptomic datasets generated and analyzed in this study are publicly available in the Gene Expression Omnibus (GEO) under the accession numbers referenced in the manuscript. In addition, the mouse tissue RNA-seq data generated in this study have been deposited in the GEO database under accession number GSE307343.
