# Supplementary material for: Loss of Pum2 exacerbates colitis by disrupting macrophage–epithelial crosstalk and promoting epithelial necroptosis
Source: Cell Death Discov. 2026 Mar 20;12:137. doi: 10.1038/s41420-026-03041-x (PMC13039920; doi:10.1038/s41420-026-03041-x)
Supplement: Supplementary file 1 — Supplementary Methods [file 41420_2026_3041_MOESM1_ESM.docx]

**Supplementary Methods**

**1. Acquisition and analysis of transcriptomic data**

To assess Pum2 expression across gastrointestinal inflammatory and neoplastic conditions, publicly available transcriptomic datasets were retrieved from the NCBI Gene Expression Omnibus (GEO). Datasets included patients with inflammatory bowel disease (IBD), ulcerative colitis (UC), Crohn’s disease (CD), and colorectal cancer (CRC). Murine datasets were included to complement human findings and to capture Pum2 dynamics under controlled experimental paradigms of colitis and tumorigenesis. The following GEO datasets were analyzed: GSE193677, GSE186582, GSE16879, GSE4183, GSE42768, GSE166708, GSE43338, and GSE148794. Data processing, normalization, and differential expression analyses were performed in R v4.2.0 and relevant Bioconductor packages. Group comparisons were defined according to metadata annotations. In addition, ORF-based datasets (GSE175685, GSE186110) were examined to identify upstream regulators of Pum2 expression in THP-1 and HT-29 cells.

**2. Dataset selection and eligibility**

For IBD and related conditions: (("inflammatory bowel diseases"[MeSH Terms] OR inflammatory bowel disease[All Fields]) AND IBD[All Fields]) AND "Homo sapiens"[All Fields] AND ("1000"[n_samples] : "10000000"[n_samples]). This search retrieved 3 datasets. Among them, only GSE193677 provided transcriptomic data from multiple intestinal regions, including the colon and rectum, and was selected for further analysis.

For therapeutic cohorts: "("inflammatory bowel diseases"[MeSH Terms] OR inflammatory bowel disease[All Fields]) AND IBD[All Fields]) AND "patients"[All Fields] AND "treatment"[All Fields] AND ("100"[n_samples] : "10000000"[n_samples]). This search identified 17 datasets. After applying the exclusion criteria (i.e., removing 2 pediatric IBD datasets, 2 involving atypical IBD patient data, 2 single-cell RNA-seq datasets, 2 datasets without paired pre- and post-treatment samples, 1 genomic methylation dataset, 2 blood-based datasets, and 5 datasets lacking relevant annotations), GSE16879 was retained. This dataset includes paired pre-treatment and post-treatment samples from patients, meeting our criteria. For long-term therapeutic response studies, we further applied the same exclusion criteria to select datasets with paired pre- and post-treatment data and the longest observation periods, thus enabling the analysis of therapeutic dynamics over extended durations.

For animal models (acute and chronic DSS models): ("acute"[All Fields] AND "chronic"[All Fields] AND "DSS"[All Fields]) AND "Mus musculus"[porgn] AND ("attribute name tissue"[Filter] AND ("20"[n_samples] : "10000000"[n_samples])). This search identified 5 datasets. We excluded 2 datasets containing macrophage-derived data, 1 macro-RNA sequencing dataset, and 1 dataset with no available sequencing annotations. The dataset GSE42768 was retained, as it met the inclusion criteria.

Moreover, these datasets captured both inflammatory and neoplastic contexts in humans and mice, such as GSE4183, GSE166708, and GSE43338, which enabled the analysis of the inflammation-cancer transition. These datasets provide valuable insights into the pathophysiological relationship between chronic inflammation and cancer development in both animal models and human subjects. Additionally, two ORF-based datasets (GSE175685, GSE186110) were analyzed separately to explore upstream regulation of Pum2 in THP-1 and HT-29 cells.

**3. Mouse models**

Pum2-knockout (Pum2−/−) mice were kindly provided by Professor Eugene Yujun Xu (Nanjing Medical University) [1] and maintained on an FVB background through serial backcrossing with wild-type FVB mice. Genotypes were confirmed by PCR, and knockout efficiency was validated by qPCR and IHC. All mice were housed under specific pathogen-free (SPF) conditions at the Animal Resource Centre of Tongji Medical College, Huazhong University of Science and Technology. All animal experiments were approved by the Institutional Animal Care and Use Committee (IACUC No. 4349) of Huazhong University of Science and Technology.

**4. Dextran sulfate sodium (DSS)-induced colitis model**

To investigate the role of Pum2 in colonic inflammation, age-matched male WT and Pum2−/− mice (8–10 weeks) were subjected to DSS-induced colitis. Untreated mice of both genotypes were included as baseline controls. Experiments were conducted in two independent cohorts under identical conditions, with consistent results. To reduce microbiota-related variability and cage effects, WT and Pum2−/− littermates were cohoused or randomly assigned to cages in the same SPF facility, with autoclaved chow and sterile water. Animals from multiple cages were included per group, and analyses were performed at the individual level.

Mice received 2.5% DSS (molecular weight 36,000–50,000; MP Biomedicals) dissolved in drinking water continuously for nine days, followed by a three-day recovery period with regular water. Throughout the experimental course, clinical indicators—including body weight, stool consistency, and the presence of rectal bleeding—were monitored daily. Disease severity was quantified using a standardized Disease Activity Index (DAI) scoring system ranging from 0 to 4, where 0 indicated normal stool without bleeding, and 4 corresponded to liquid diarrhea with overt rectal hemorrhage [2]. At the study endpoint (day 12), mice were euthanized, and colons were excised from the rectum to the cecum. The study was performed in two independent cohorts under identical DSS conditions to ensure reproducibility. Outcome assessments (clinical scoring, histology, and image quantification) were performed in a blinded manner. Data shown are from one representative cohort (n = 8 per group at endpoint), while survival analyses prospectively pooled both cohorts (n = 25 per group).

**5. Cell culture and treatment protocols**

Primary bone marrow-derived macrophages (BMDMs) were harvested from the femoral and tibial bones of either wild-type or Pum2 knockout (Pum2−/−) mice and cultured in α-MEM supplemented with 30 ng/mL M-CSF, 10% FBS, and 1% penicillin–streptomycin. RAW264.7 murine macrophages were maintained in DMEM with 10% FBS and 1% penicillin–streptomycin. Macrophage polarization toward the M1 phenotype was induced by treatment with LPS (100 ng/mL) and IFNγ (25 ng/mL) for 24 h. Caco2 cells were cultured in RPMI 1640 supplemented with 10% FBS and 1% penicillin–streptomycin. For inflammatory activation assays, cells were treated with LPS (100 ng/mL) for 30 min or 24 h, depending on the experimental design.

**6. Generation of Pum2−/− BMDMs and Pum2 overexpression cell lines**

Pum2 knockout (Pum2−/−) bone marrow-derived macrophages (BMDMs) were obtained from Pum2 KO mice. To generate Pum2-overexpressing cell lines, 293T cells cultured in 10-cm dishes were co-transfected with 8 μg of a Pum2-expressing plasmid (oePum2, Vigenebio) or empty vector (Vigenebio), 8 μg of psPAX2, and 4 μg of pMD2.G (Shanghai Genechem) using Lipomaster 3000 Transfection Reagent (Nanjing Vazyme Biotech), following the manufacturer’s protocol. After 48–72 hours, lentiviral supernatants were collected and concentrated by ultracentrifugation. The viral particles were then used to transduce RAW264.7, THP-1, and Caco2 cells in the presence of a viral transduction enhancer (Shanghai Genechem, HitransG P). For suspension THP-1 cells, infection was performed at 1,000 rpm for 1 hour to enhance transduction efficiency. Stably infected cells were selected with puromycin (4 μg/mL, Biosharp).

**7. Transwell co-culture system to model macrophage–epithelial interactions**

A Transwell co-culture setup featuring 0.4 μm pore polycarbonate membranes (Corning, NY, USA) was employed to examine intercellular communication between macrophages and epithelial cells. Human THP-1 monocytes were differentiated into macrophages through treatment with 100 nM phorbol 12-myristate 13-acetate (PMA) for 48 hours in 6-well plates. Simultaneously, Caco2 cells were seeded on the upper chamber inserts to establish a confluent epithelial monolayer. THP-1-derived macrophages were then incubated in media with or without LPS (100 ng/mL, Sigma-Aldrich), in the presence or absence of a neutralizing anti-human TNFα antibody (5 μg/mL; Sigma-Aldrich) to selectively inhibit TNFα activity. Following a 24-hour co-culture period, cellular and supernatant samples were collected for downstream analyses.

**8. Histopathological assessment and immunohistochemical analysis**

Formalin-fixed, paraffin-embedded colonic tissues were sectioned at 4 μm thickness and mounted on glass slides. For histopathological evaluation, sections were deparaffinized, rehydrated through graded ethanol, and stained with hematoxylin and eosin (H&E) following standard procedures. Tissue sections were examined in a blinded manner by two independent investigators. The severity of inflammation was graded on a 0–4 scale based on histological criteria: 0, no inflammatory changes; 1, mild inflammation with scattered mononuclear cell infiltration (1–2 foci); 2, moderate inflammation involving multiple foci; 3, severe inflammation characterized by increased vascular density and marked thickening of the intestinal wall; 4, maximal inflammatory response with transmural leukocyte infiltration and depletion of goblet cells[3].

For immunohistochemistry (IHC), tissue sections underwent deparaffinization and rehydration, followed by heat-induced antigen retrieval in citrate buffer (pH 6.0) using a microwave oven for 10 minutes. Endogenous peroxidase activity was blocked with 3% hydrogen peroxide for 10 minutes at room temperature. Sections were then incubated with 5% normal goat serum for 30 minutes to reduce nonspecific binding. Primary antibodies used included anti-iNOS (Proteintech, 1:200), Pum2 (Abcam, 1:200), F4/80 (Santa Cruz Biotechnology, 1:300), CD3 (Servicebio, 1:500), CD4 (Servicebio, 1:400), and CD8 (Servicebio, 1:1000), applied overnight at 4 °C in a humidified chamber. After washing, sections were incubated with HRP-conjugated secondary antibodies (Servicebio) and visualized using a DAB substrate kit (Servicebio). Hematoxylin was used for nuclear counterstaining. Slides were dehydrated, mounted, and examined under a bright-field microscope.

**9. Immunofluorescence staining**

Cells were fixed with 4% paraformaldehyde and permeabilized using 0.05% Triton X-100. After blocking with 5% bovine serum albumin (BSA) for 30 minutes, cells were incubated overnight at 4 °C with primary antibodies against TNFα (Proteintech, 1:200) and iNOS (Proteintech, 1:200). After washing, cells were incubated for 1 hour at room temperature in the dark with fluorophore-conjugated secondary antibodies, including Cy3–conjugated Goat Anti-Rabbit IgG (H+L) (Proteintech).

Intracellular ROS in colonic tissues was assessed using the fluorescent probe DCFH-DA (10 μM, Beyotime, China). For mitochondrial ROS detection, MitoSOX™ Red (Beyotime) was used according to the manufacturer’s instructions.

For tissue staining, paraffin-embedded mouse colonic sections were processed as previously described[4]. After antigen retrieval and blocking, sections were incubated with antibodies against ZO1 (Proteintech, 1:200), Occludin (Proteintech, 1:200), Ki67 (Servicebio, 1:300), TNFα (Proteintech, 1:200). ZO1 and Occludin staining were detected using CoraLite488–conjugated Goat Anti-Rabbit IgG (H+L) (Proteintech), TNFα was detected using Cy3–conjugated Goat Anti-Rabbit IgG (H+L) (Proteintech), and Ki67 was detected using Cy3–conjugated Goat Anti-Mouse IgG (H+L) (Proteintech).

Apoptotic cells were detected using a TUNEL assay kit (Servicebio, Wuhan, China) following the manufacturer’s protocol. Fluorescence images were acquired with a Nikon confocal microscope.

**10. Flow cytometry**

Surface marker expression on BMDMs and RAW264.7 cells was analyzed by flow cytometry. Cells (1 × 10⁶ per sample) were harvested, washed with PBS containing 2% FBS, and incubated with PE-conjugated anti-F4/80 and APC-conjugated anti-CD86 antibodies (BioLegend) for 30 minutes at 4 °C in the dark. After staining, cells were washed and resuspended in PBS.

For ROS detection, cells were washed with serum-free medium and incubated with the membrane-permeable fluorescent probe DCFH-DA (10 μM, Beyotime, China) at 37 °C for 20 minutes in the dark, according to the manufacturer's instructions. As DCFH-DA freely diffuses across the cell membrane, no membrane permeabilization was required. After incubation, cells were washed with PBS to remove excess probe, collected, and immediately subjected to flow cytometric analysis.

Data acquisition was performed on a BD FACSymphony™ A1 flow cytometer (BD Biosciences), and results were analyzed using FlowJo software.

**11****. Western blot analysis**

Whole-cell protein extracts were prepared in RIPA buffer supplemented with protease and phosphatase inhibitors. Equal amounts of protein (20–40 μg) were separated by SDS-PAGE and transferred onto PVDF membranes (Millipore). After blocking with 5% non-fat milk in TBST for 1 hour at room temperature, membranes were incubated overnight at 4 °C with primary antibodies against Pum2 (Abcam), TNFα (Proteintech), phospho-RIPK1 (Ser166), phospho-RIPK3 (Ser227), phospho-MLKL (Ser358) (all from Abmart), ZO1 and Occludin (Proteintech), NOX2 (Chengdu Zhengneng Biotechnology), GAPDH (Proteintech), and β-actin (Cell Signaling Technology). After incubation with HRP-conjugated secondary antibodies, bands were visualized using enhanced chemiluminescence (ECL, biosharp) and imaged with a ChemiDoc Touch Imaging System (Bio-Rad).

**12. Statistical analysis**

All data are presented as the mean ± standard deviation (SD). Comparisons between two groups were performed using paired or unpaired Student’s t-tests, as appropriate. Multiple group comparisons were conducted by one-way ANOVA, followed by Dunnett’s post hoc test (for comparisons against a single control) or Tukey’s multiple comparisons test (for all pairwise comparisons). For non-parametric data, the Mann–Whitney U test or Kruskal–Wallis test with Dunn’s post hoc correction was applied. Kaplan–Meier survival curves were analyzed using the log-rank test. All statistical analyses were performed using GraphPad Prism v8.0 and R v4.2.0. A P value < 0.05 was considered statistically significant.

**References**

1. Lin, K., Qiang, W., Zhu, M., Ding, Y., Shi, Q., Chen, X., Zsiros, E., Wang, K., Yang, X., Kurita, T. & Xu, E. Y. Mammalian Pum1 and Pum2 Control Body Size via Translational Regulation of the Cell Cycle Inhibitor Cdkn1b. Cell Rep, 2019; 26:2434-2450.e2436. <https://doi.org:10.1016/j.celrep.2019.01.111>

2. Poddar, D., Kaur, R., Baldwin, W. M., 3rd & Mazumder, B. L13a-dependent translational control in macrophages limits the pathogenesis of colitis. Cell Mol Immunol, 2016; 13:816-827. <https://doi.org:10.1038/cmi.2015.53>

3. Wirtz, S. & Neurath, M. F. Mouse models of inflammatory bowel disease. Adv Drug Deliv Rev, 2007; 59:1073-1083. <https://doi.org:10.1016/j.addr.2007.07.003>

4. Liu, C., Gan, Y. H., Yong, W. J., Xu, H. D., Li, Y. C., Hu, H. M., Zhao, Z. Z. & Qi, Y. Y. OTUB1 regulation of ferroptosis and the protective role of ferrostatin-1 in lupus nephritis. Cell Death Dis, 2024; 15:791. <https://doi.org:10.1038/s41419-024-07185-5>
