# Supplementary material for: Loss of Pum2 exacerbates colitis by disrupting macrophage–epithelial crosstalk and promoting epithelial necroptosis
Source: Cell Death Discov. 2026 Mar 20;12:137. doi: 10.1038/s41420-026-03041-x (PMC13039920; doi:10.1038/s41420-026-03041-x)
Supplement: Supplementary file 2 — Supplementary Figures 1-6. [file 41420_2026_3041_MOESM2_ESM.docx]

**
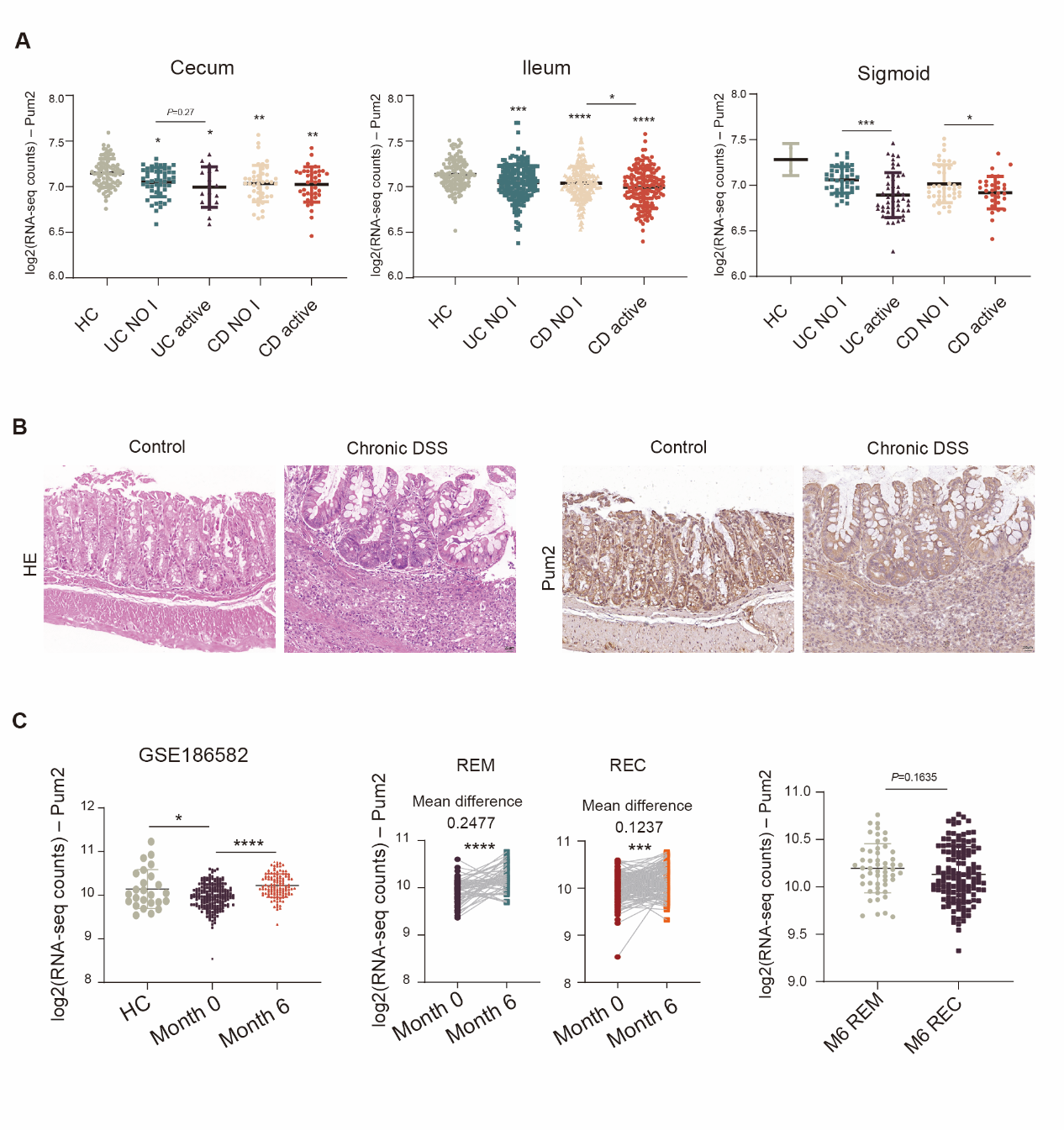
**

**Supplementary Figure 1. Additional characterization of Pum2 dynamics in human colitis.**

**(A)** Pum2 expression in cecum, ileum, and sigmoid biopsies (GSE193677). A downward trend was observed in UC/CD patients compared with healthy controls. One-way ANOVA with Tukey’s post-hoc test. *P < 0.05; **P < 0.01; ***P < 0.001; ****P < 0.0001. HC: healthy controls; UC NO I: non-inflamed UC patients; UC active: active UC patients; CD NO I: non-inflamed CD patients; CD active: active CD patients.

**(B)** H&E staining and Pum2 IHC staining of chronic DSS-treated colon. The chronic DSS protocol involved administering 2.5% DSS in drinking water for 7 days, followed by 14 days of regular water, with three cycles of DSS treatment. Left: H&E staining revealed typical features of DSS-induced chronic colitis, including crypt distortion, loss of goblet cells, mucosal ulceration, colonic fibrosis, and epithelial dysplasia. Right: Pum2 IHC staining showed downregulation of Pum2 in the chronic DSS-treated colon. Scale bar = 20 μm.

**(C)** Time-course analysis of Pum2 expression in IBD patients (GSE186582) before (Month 0, M0) and after six-month therapy (Month 6, M6). Left: before vs after treatment (one-way ANOVA, Tukey’s post hoc). Middle: within-group comparison (Month 0 vs Month 6) in remission (REM) and relapse (REC) cohorts (paired t-test). Right: post-treatment (M6) comparison between REM and REC (unpaired t-test). *P < 0.05; **P < 0.01; ****P < 0.0001. HC: healthy controls; M0: baseline (before treatment); M6: after 6 months of treatment.


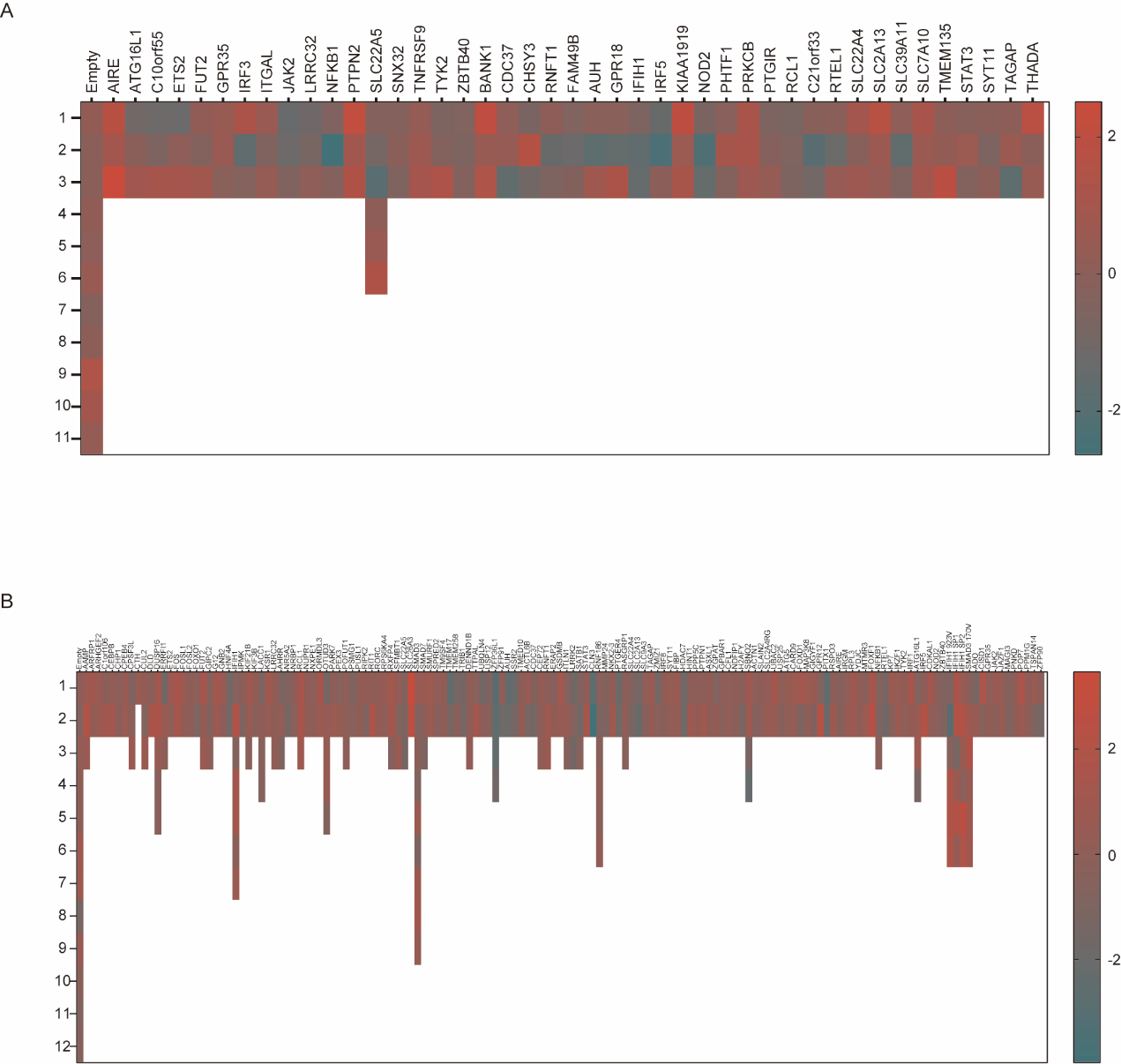


**Supplementary Figure 2. Pum2 expression profiling in response to IBD-associated gene overexpression.**

**(A)** Heatmap of Pum2 expression in THP-1 cells transduced with open reading frames (ORFs) corresponding to IBD susceptibility genes (GSE175685).

**(B)** Heatmap of Pum2 expression changes in HT-29 cells following ORF-mediated overexpression of IBD-associated genes (GSE186110).


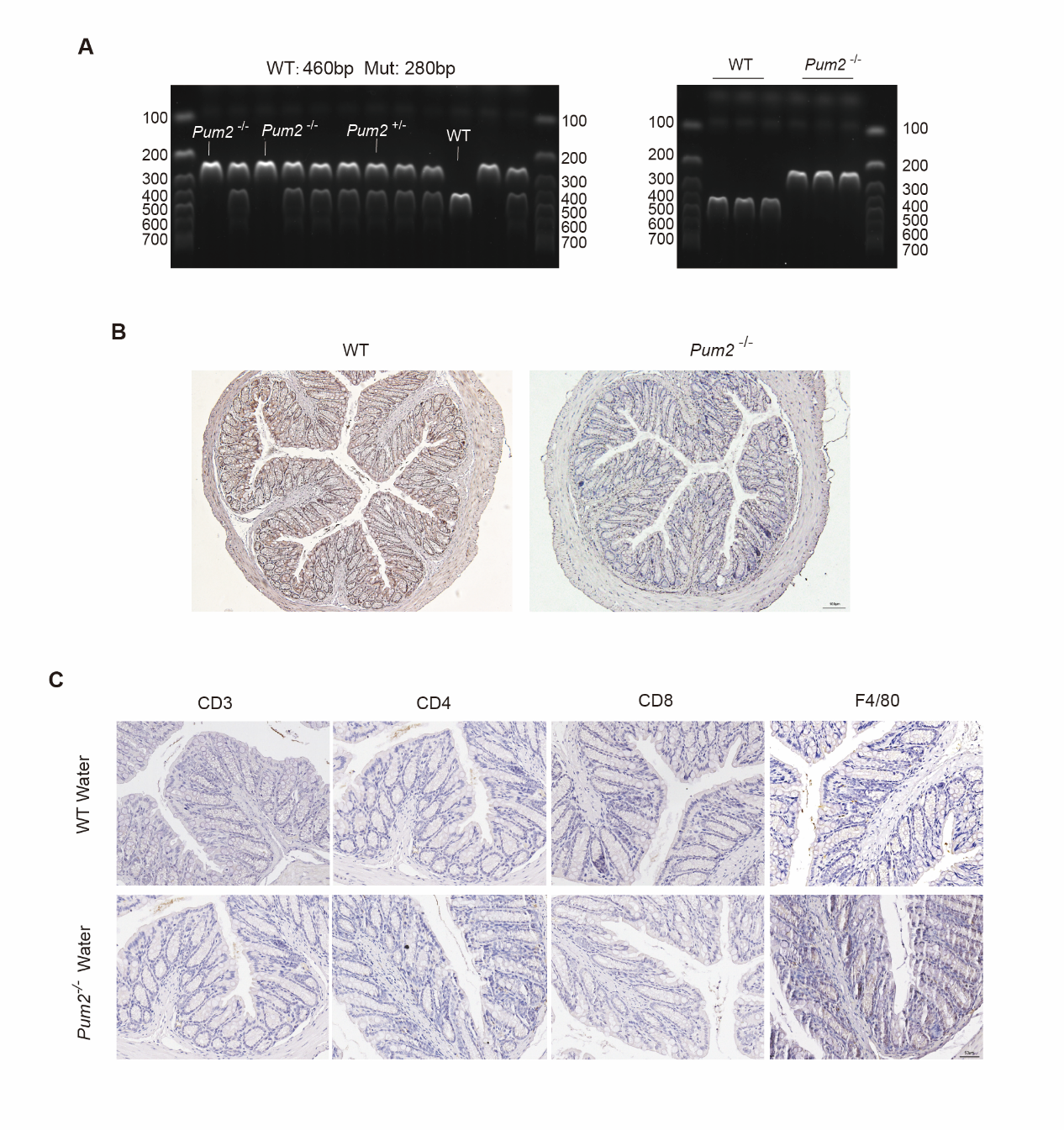


**Supplementary Figure 3. Validation of homozygous Pum2 knockout and IHC analysis**

**(A)** Genotyping of Pum2-deficient mice by PCR using tail genomic DNA. The WT allele produced a 460 bp band, whereas the mutant allele yielded a 280 bp fragment.

**(B)** IHC staining of colon tissue confirmed loss of Pum2 protein in Pum2−/− colons compared with WT controls. Scale bar = 100 μm.

**(C)** IHC staining of colonic tissues from WT water and Pum2−/− water groups, stained for CD3, CD4, CD8, and F4/80 to evaluate T-cell and macrophage infiltration. Scale bar = 50 μm.


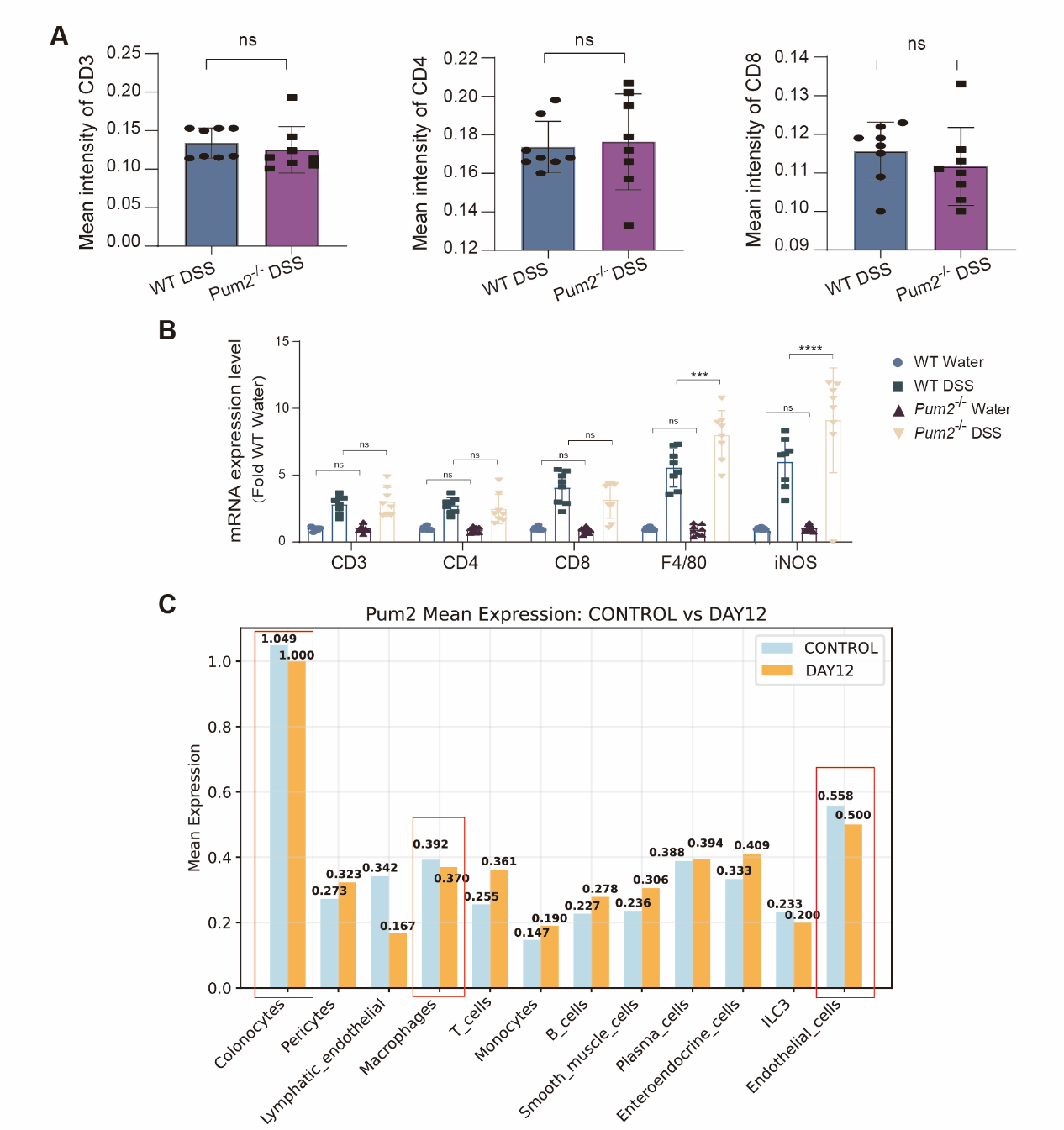


**Supplementary Figure 4. Infiltration of immune cell subsets in colonic tissues following DSS challenge.**

**(A)** IHC staining of CD3+, CD4+, and CD8+ T cell populations in colon sections from WT and Pum2−/− mice after DSS treatment (n = 8). Statistical analysis was performed using unpaired t-test. No statistically significant differences were observed (ns, not significant).

**(B)** qPCR analysis of CD3, CD4, CD8, F4/80, and iNOS expression in colons from WT water, WT DSS, Pum2-/- water, and Pum2-/- DSS groups. One-way ANOVA with Tukey’s post hoc. ns: not significant; ***P < 0.001; ****P < 0.0001.

**(C)** Single-cell RNA-seq (GSE148794) showing Pum2 expression across colonic cell populations under basal and DSS-induced conditions (Day 12: 6 days DSS + 6 days water recovery).


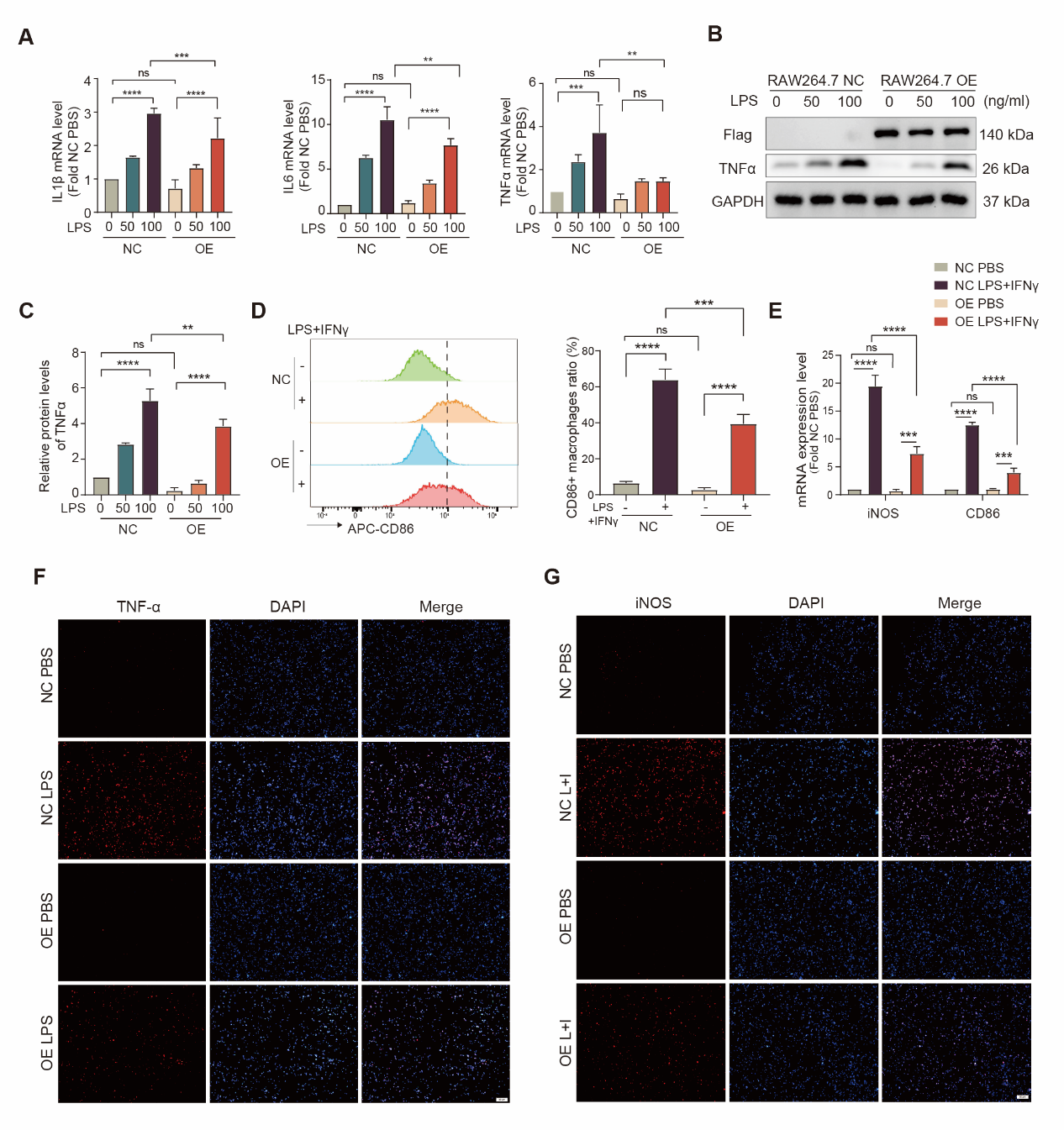


**Supplementary Figure 5. Pum2 overexpression suppresses macrophage inflammatory activation and cytokine production**

**(A)** RT-qPCR of IL1β, IL6, and TNFα in RAW 264.7 macrophages overexpressing Pum2 (OE-Pum2) or negative control (OE-NC), under basal or LPS stimulation (100 ng/mL, 6 h). One-way ANOVA with Tukey’s post hoc. ns: not significant; **P < 0.01; ***P < 0.001; ****P < 0.0001. NC: OE-NC RAW 264.7 cells; OE: OE-Pum2 RAW 264.7 cells.

**(B-C)** Western blot of TNFα protein in OE-Pum2 and OE-NC macrophages treated with LPS (100 ng/mL, 6 h) and brefeldin A (1 μg/mL, 3 h). One-way ANOVA with Tukey’s post hoc. ns: not significant; *P < 0.05; **P < 0.01; ***P < 0.001; ****P < 0.0001.

**(D)** Flow cytometric analysis of CD86 expression as a marker of M1 polarization in RAW 264.7 cells following LPS/IFN-γ (24 h). One-way ANOVA with Tukey’s post hoc. ns: not significant; ***P < 0.001; ****P < 0.0001. NC: OE-NC RAW 264.7 cells; OE: OE-Pum2 RAW 264.7 cells.

**(E)** Quantification of M1 signature genes (CD86, iNOS) in OE-NC and OE-Pum2 macrophages. One-way ANOVA with Tukey’s post hoc. ns: not significant; ***P < 0.001; ****P < 0.0001.

**(F)** Immunofluorescence staining of TNFα in OE-NC and OE-Pum2 macrophages after PBS or LPS (100 ng/mL, 6 h) with BFA (1 μg/mL, 3 h).

**(G)** Representative immunofluorescence of iNOS following LPS/IFNγ stimulation (24 h).


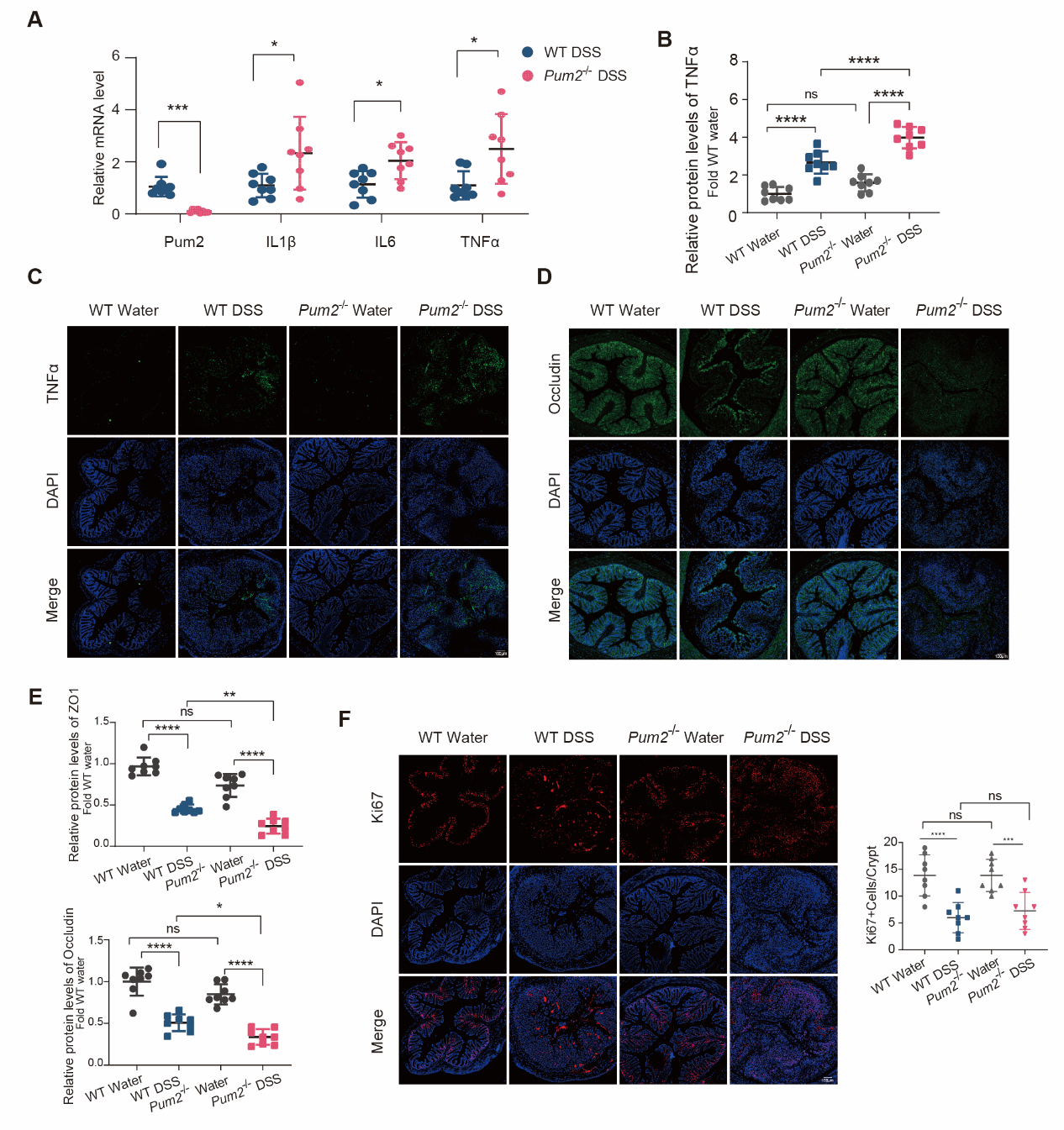


**Supplementary Figure 6. Pum2 deficiency promotes cytokine hypersecretion, TNFα accumulation, and epithelial injury in DSS-induced colitis.**

**(A)** RT-qPCR of TNFα, IL6, and IL1β in colons from DSS-treated WT and Pum2-/- mice (n = 8). Unpaired t-test. *P < 0.05; ***P < 0.001.

**(B)** Western blot quantification of TNFα in DSS-treated colons. One-way ANOVA with Tukey’s post hoc. ns: not significant; ****P < 0.0001.

**(C)** Immunofluorescence staining of TNFα (green) in colon sections after DSS. Scale bar = 100 μm.

**(D)** Immunofluorescence staining of Occludin (green) to visualize tight junction integrity. Scale bar = 100 μm.

**(E)** Western blot quantification of ZO1 and Occludin in colonic tissues (n = 8). One-way ANOVA with Tukey’s post hoc. ns: not significant; *P < 0.05; ***P < 0.001; ****P < 0.0001.

**(F)** Immunofluorescence staining of Ki67 (red) illustrating epithelial proliferation in WT and Pum2-/- colonic tissues following either water or DSS challenge. scale bar = 20 μm. Quantification of Ki67⁺ cells per crypt was performed by one-way ANOVA with Tukey’s post hoc. ns: not significant; ***P < 0.001; ****P < 0.0001.
