# Supplementary figures and images for: Loss of Pum2 exacerbates colitis by disrupting macrophage–epithelial crosstalk and promoting epithelial necroptosis
Source: Cell Death Discov. 2026 Mar 20;12:137. doi: 10.1038/s41420-026-03041-x (PMC13039920; doi:10.1038/s41420-026-03041-x)

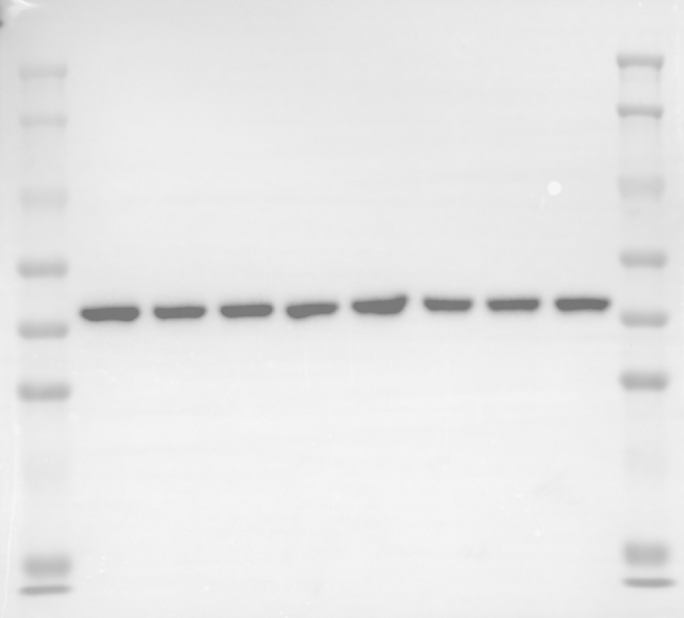

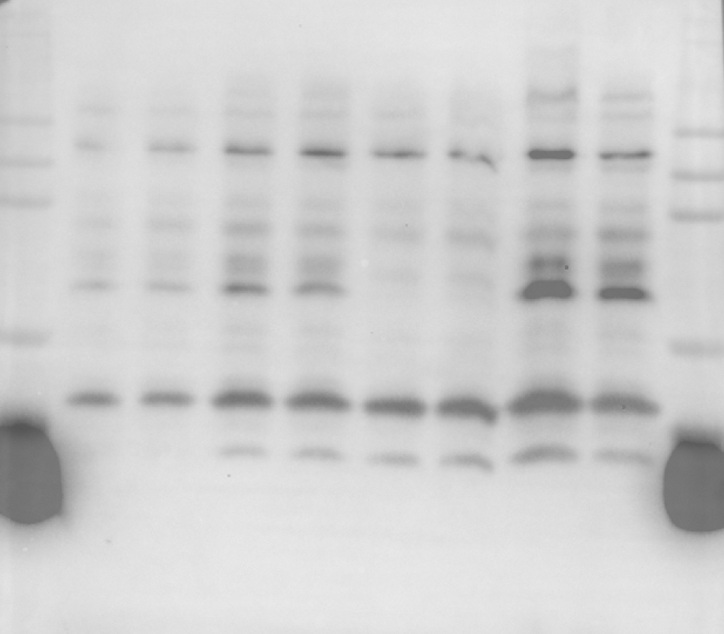

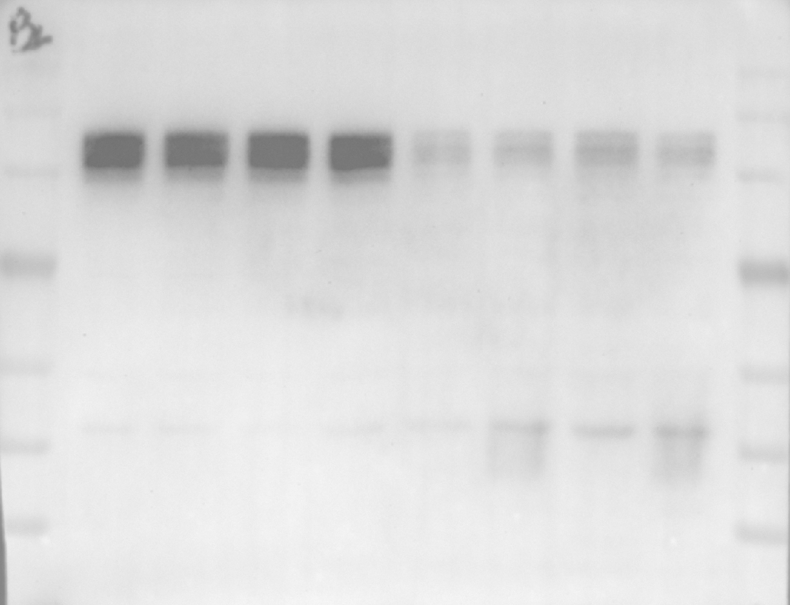


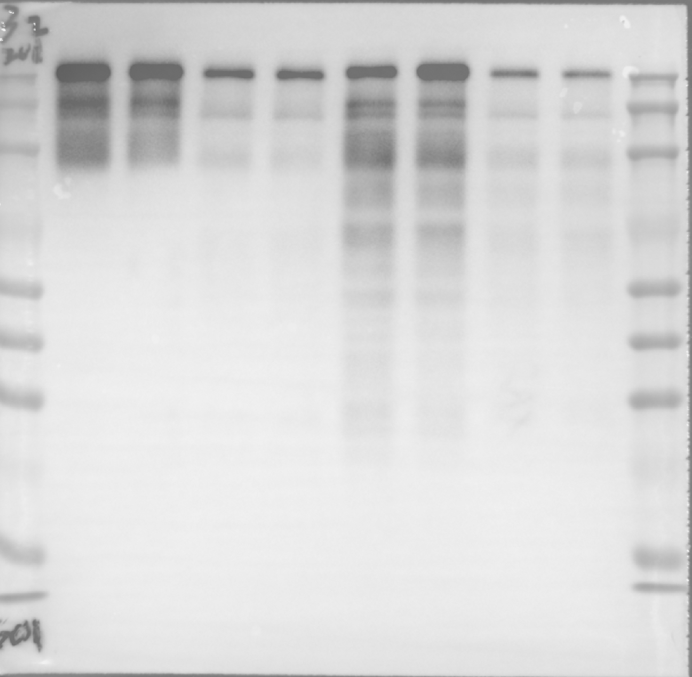

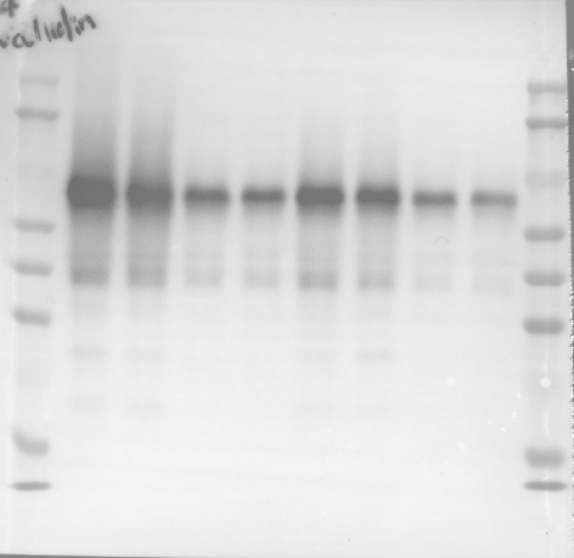

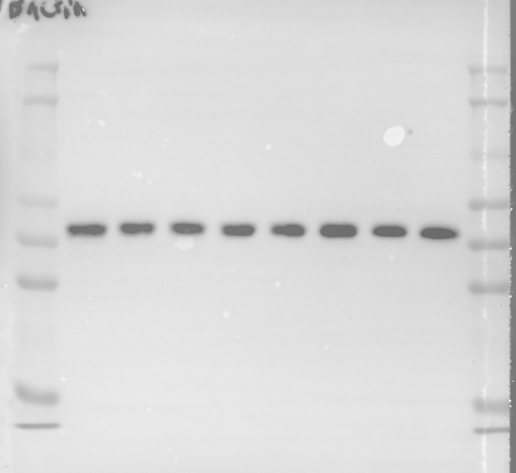


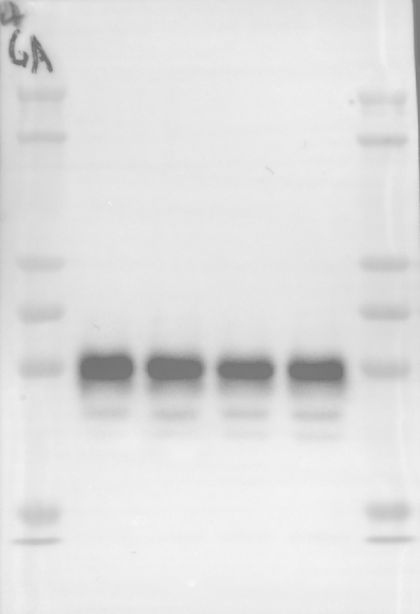

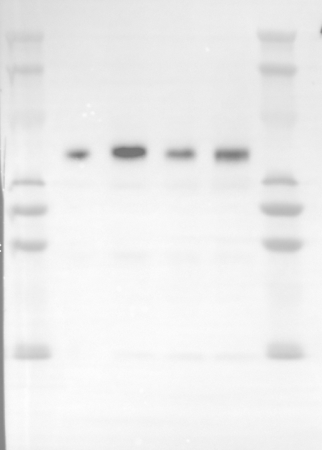


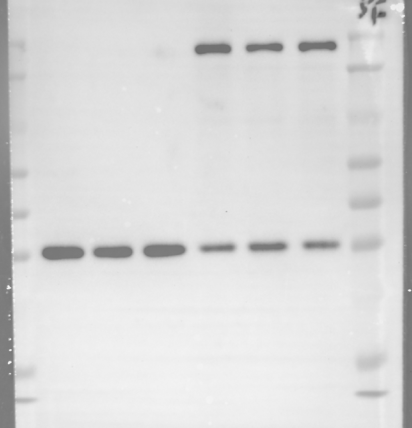

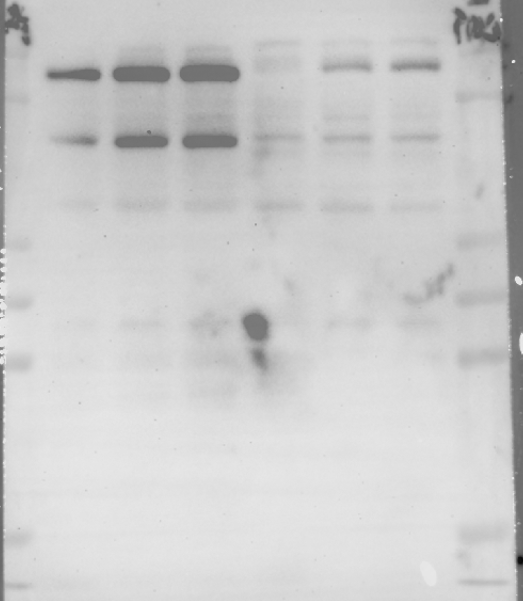

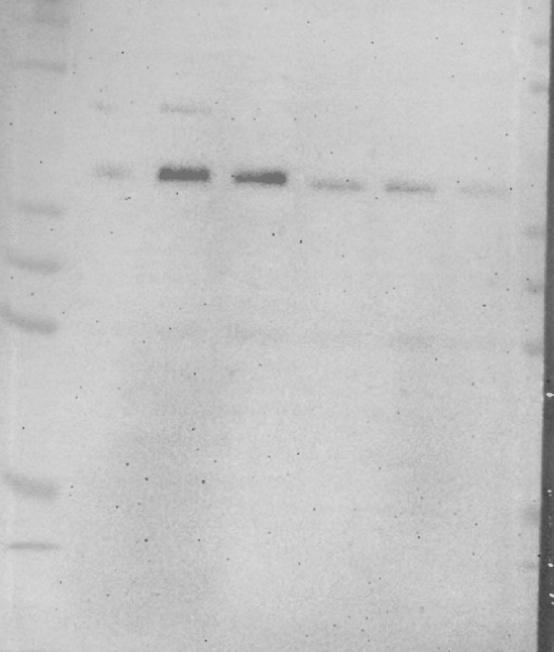


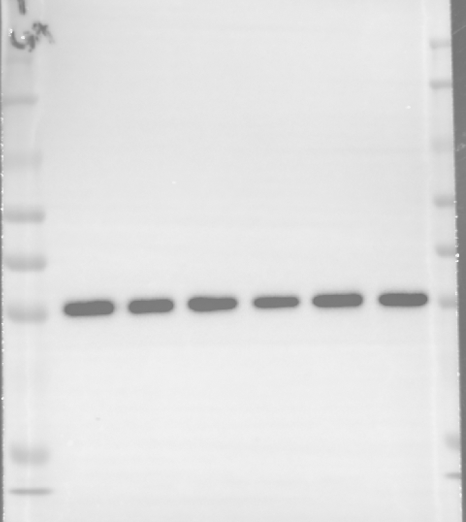


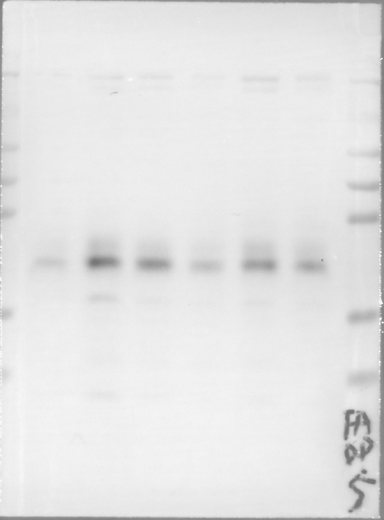

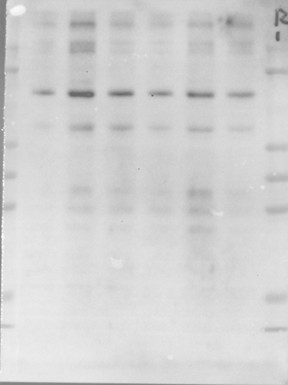

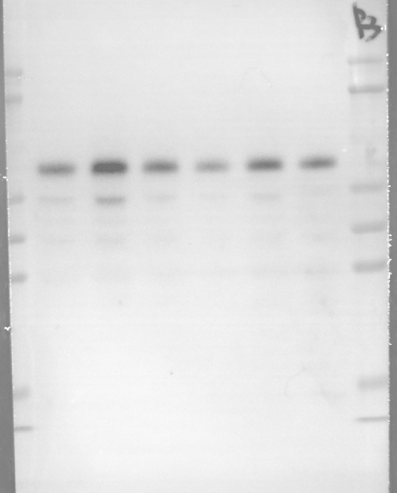


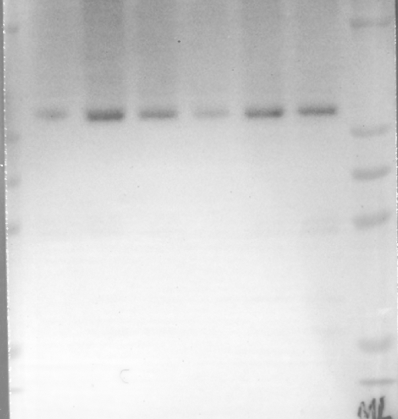

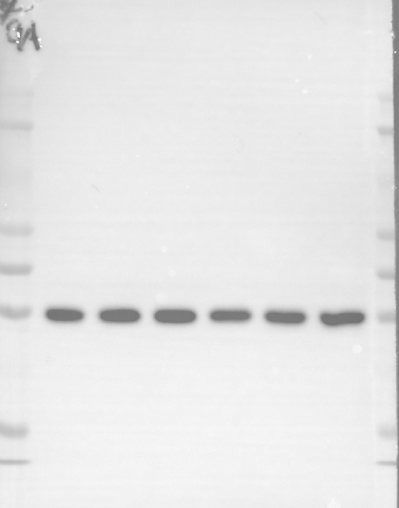


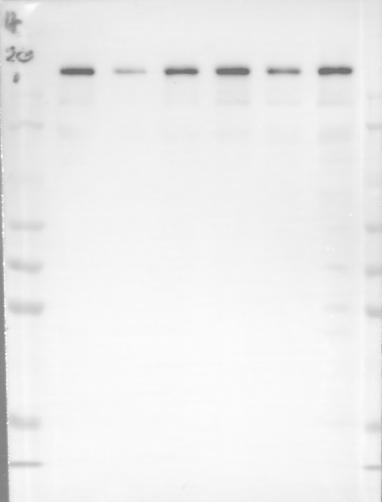

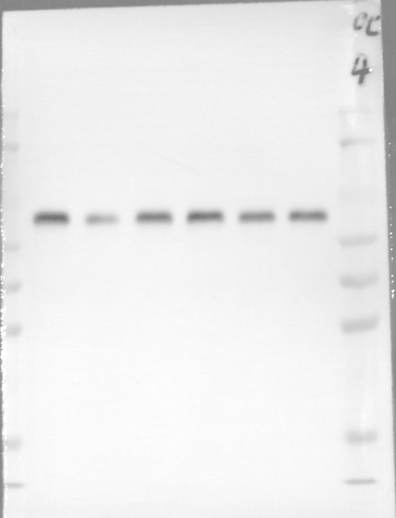

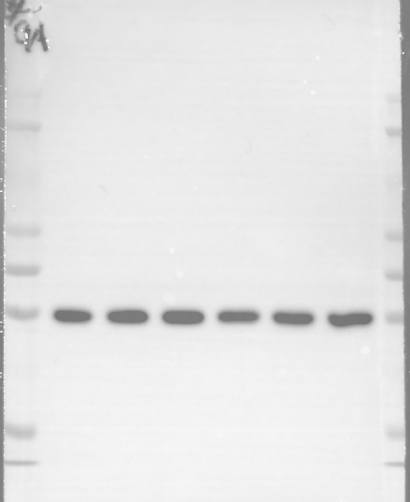


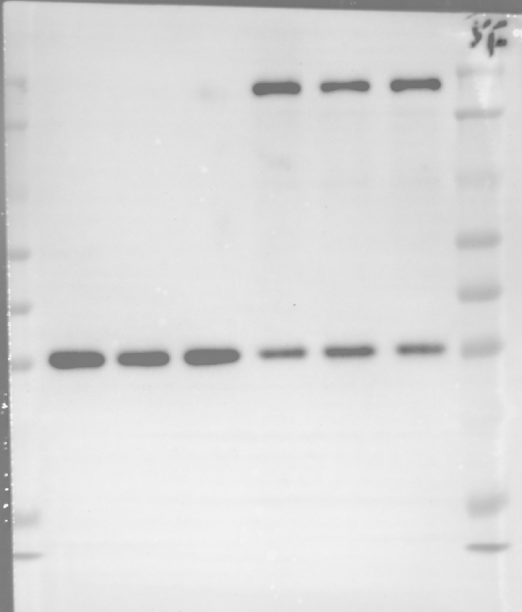

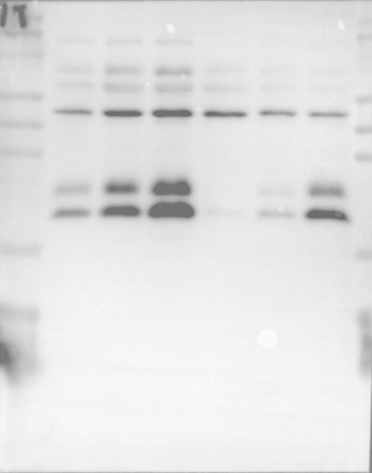

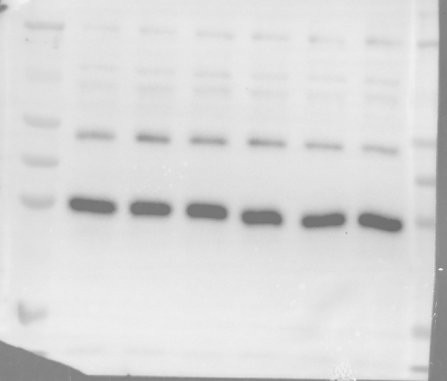

Supplement: Supplementary file 5 — WB raw data [file 41420_2026_3041_MOESM5_ESM.docx]
